# ﻿New species and records of limpets (Mollusca, Gastropoda) from the Pacific Costa Rica Margin

**DOI:** 10.3897/zookeys.1214.128594

**Published:** 2024-10-09

**Authors:** Melissa J. Betters, Jorge Cortés, Erik E. Cordes

**Affiliations:** 1 Department of Biology, Temple University, Philadelphia, PA, USA Temple University Philadelphia United States of America; 2 (CIMAR) Universidad de Costa Rica, San José, Costa Rica Universidad de Costa Rica San José Costa Rica

**Keywords:** Biodiversity, chemosynthetic ecosystems, cold seeps, deep sea, systematics, taxonomy

## Abstract

The ocean remains a reservoir of unknown biodiversity, particularly in the deep sea. Chemosynthesis-based ecosystems, such as hydrothermal vents and hydrocarbon seeps, host unique and diverse life forms that continue to be discovered and described. The present study focuses on patelliform gastropods (limpets) collected from Pacific Costa Rica Margin hydrocarbon seeps during three research cruises from 2017 to 2019. Genetic and morphological analyses revealed the presence of several new lineages within the genera *Bathyacmaea* Okutani, Tsuchida & Fujikura,1992, *Cocculina* Dall, 1882, *Paralepetopsis* McLean, 1990, and the family Lepetodrilidae McLean, 1988: *Bathyacmaealevinae***sp. nov.**, *Paralepetopsisvariabilis***sp. nov.**, *Pseudolepetodriluscostaricensis***gen. et sp. nov.**, and *Cocculinamethana***sp. nov.** These investigations also expanded the known ranges of the species *Pyropeltacorymba* McLean, 1992 and *Lepetodrilusguaymasensis* McLean, 1988 to the Costa Rica Margin. This research highlights the uniqueness of gastropod fauna at the Costa Rica Margin and contributes to our understanding of the biodiversity at chemosynthesis-based deep-sea ecosystems in the face of global biodiversity loss and increased commercial interest in deep-sea resources.

## ﻿Introduction

Despite marine species across all phyla being described at an average rate of more than 2,000 new species per year (Bouchet et al. 2023), estimates still place the unknown number of species in the ocean at approximately 0.3–2.2 million species (Mora et al. 2011; Costello et al. 2012). Species discovery in the deep ocean (defined as depths below 200 meters) tends to be slower than in other oceanic regions. Bouchet et al. (2023) examined 600 randomly selected, newly discovered marine species from around the globe and found that just 7% came from depths below 1,000 meters. Representation of the deep ocean is also consistently lower than shallow waters on biodiversity platforms like the Ocean Biodiversity Information System (OBIS; Webb et al. 2010). With increasing commercial interest in deep-sea resources (e.g., deep-sea mining; Van Dover et al. 2020), increasing attention paid to the ocean’s role in regulating climate (e.g., Levin et al. 2020), and the global threat of biodiversity loss across ecosystems (IPBES 2019), documenting marine biodiversity before it is lost is an increasingly salient issue.

Chemosynthesis-based ecosystems, such as hydrothermal vents and hydrocarbon seeps, represent biodiversity hotspots on the ocean floor (Levin et al. 2016). In the first 30 years after their initial discovery in 1977, more than 1,300 species had been described from chemosynthesis-based ecosystems (Corliss et al. 1979; German et al. 2011). Chemosynthesis, primary production fueled by fluids expelled from the seafloor through mantle dewatering processes (hydrocarbon seeps; Suess 2014) or geothermal activity (hydrothermal vents; Corliss et al. 1979), produces enough energy to support entire, diverse ecosystems (Cavanaugh et al. 1981; Levin et al. 2016). Comparatively, the rest of the deep ocean is relatively nutrient-poor (Van Dover and Trask 2000; Levin et al. 2001). Thus, many species at chemosynthesis-based ecosystems are endemic to these environments, as they are often directly or indirectly reliant on chemosynthetically derived carbon (Cordes et al. 2006; Bergquist et al. 2007; Cordes et al. 2009; German et al. 2011).

Hydrocarbon seeps at the Costa Rica Margin (CRM) host an abundance and diversity of deep-sea fauna that were extensively sampled during three cruises from 2017–2019. These sampling efforts yielded a high abundance and diversity of life, of which patelliform gastropod mollusks comprised a large portion. Patelliform gastropods (hereafter “limpets”) are common denizens at chemosynthesis-based ecosystems and are often the primary biofilm grazers at these sites (Sasaki et al. 2010 in Kiel 2010). Limpets are not monophyletic; rather, this body plan has evolved many times in the deep ocean, potentially due to its versatility in adapting to highly variable habitats (Vermeij 2017; Chen and Watanabe 2020). Current consensus on gastropod taxonomy delineates six discrete subclasses: Caenogastropoda, Heterobranchia, Neomphaliones, Neritimorpha, Patellogastropoda, and Vetigastropoda (Bouchet et al. 2017). Patellogastropoda, Neomphaliones, and Vetigastropoda are all found at chemosynthesis-based ecosystems. While several genera within these subclasses were preliminarily recognized from biological samples from the CRM, the exact species present at the CRM are largely unknown.

The present study aims to characterize the diversity of limpet species at the CRM hydrocarbon seeps. We herein investigate the genetic identities of limpet species at the CRM and whether these species are new to science. The CRM is situated near the Central American Isthmus and is separated from other nearby vent and seep fields by tens to hundreds of kilometers. Furthermore, it is positioned in the path of the equatorial currents and countercurrents, which move water west and east across the Pacific, as well as the Costa Rica Thermal Dome, which brings deep water to the surface (Kessler 2006; Fiedler and Talley 2006). This geographic position leads us to hypothesize that the CRM will host endemic species that are closely related to, but distinct from, known species from nearby regions. This paper thus undertakes the identification and description of the limpet species at the CRM and contributes novel information about the biodiversity that inhabits chemosynthesis-based ecosystems in the deep sea.

## ﻿Materials and methods

### ﻿Materials

**Figure 1.**
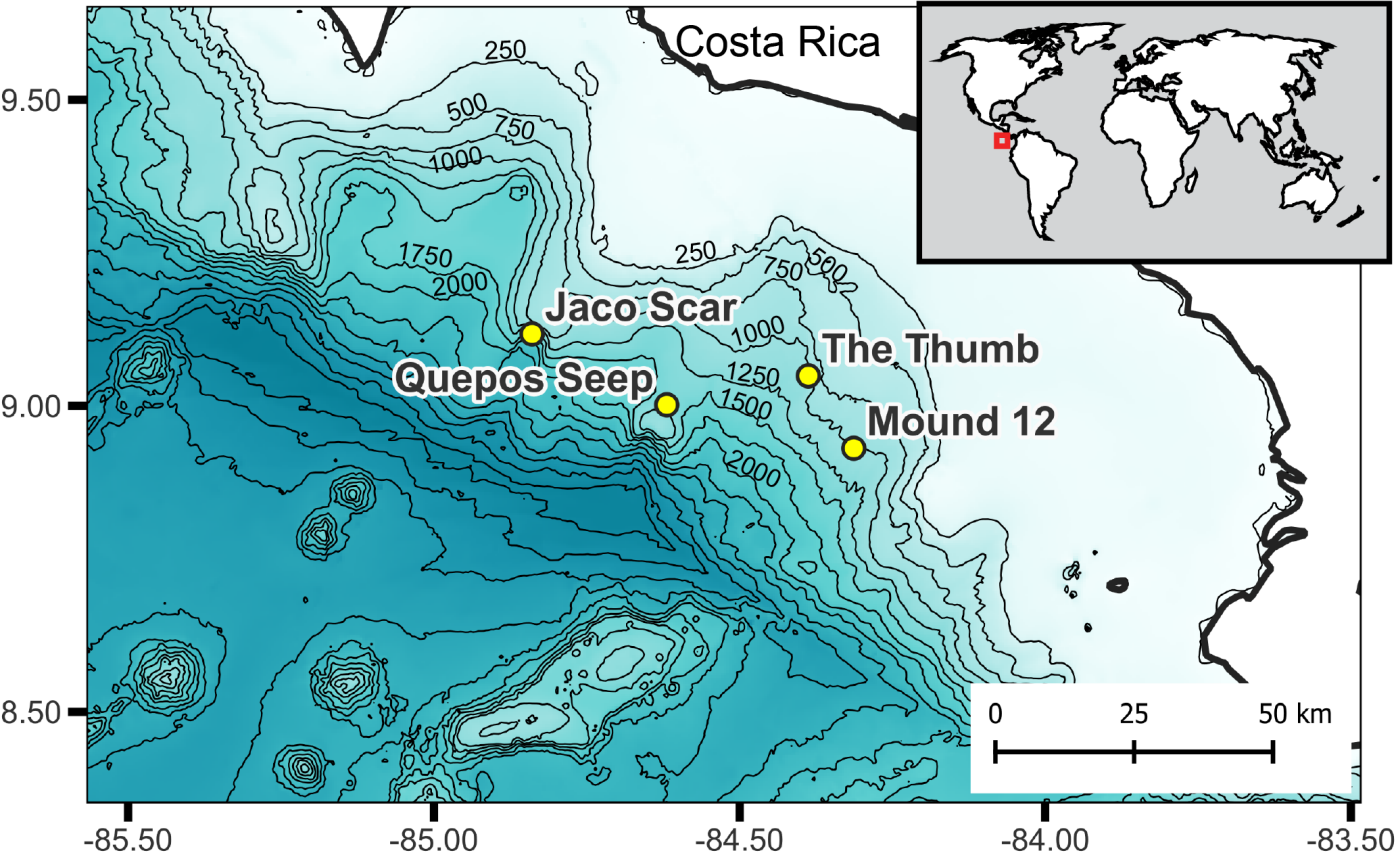
Map of the Pacific Costa Rica Margin. Four hydrocarbon seep locations from which patelliform gastropods were collected are depicted: Jaco Scar (9.12, -84.84), Quepos Seep (9.03, -84.6), The Thumb (9.05, -84.4), and Mound 12 (8.93, -84.3). Contour lines denote bottom seafloor bathymetry in meters and are drawn every 250 m.

Limpet specimens were collected from four hydrocarbon seep sites at the Pacific Costa Rica Margin (Fig. 1) during three cruises aboard the research vessels R/V Atlantis (**AT**) and R/V Falkor (**FK**): AT37-13 (Spring 2017), AT42-03 (Fall 2018), FK19-0106 (winter 2019). Additional specimens from the same hydrocarbon seep sites collected during AT15-44 (winter 2009) and AT15-59 (winter 2010) were also examined. The human-operated vehicle (HOV) Alvin was used aboard the R/V Atlantis and the remotely operated vehicle (ROV) SuBastian was used aboard the R/V Falkor. Specimens were collected in situ using sampling tools attached to the HOV and ROV such as vacuum suction and manipulator arms. Time and date of sampling events were recorded during each dive. Upon arrival to the surface, limpets were sorted into distinct shell morphotypes, promptly placed into > 95% ethanol, and subsequently stored at room temperature. All type specimens yielded are deposited at the Scripps Institute of Oceanography Benthic Invertebrate Collection (**SIO-BIC**) in San Diego, California, USA, or at the Museo de ZoologÍa de la Universidad de Costa Rica (**MZUCR**) in San José, San Pedro, Costa Rica (see the section “New species and records” below for more details on type materials). All other specimens collected are either stored at SIO-BIC, or in the personal collections of Erik Cordes at Temple University in Philadelphia, Pennsylvania, USA.

### ﻿Morphological characterization

Shells and soft tissues of all morphotypes were photographed using an AmScope microscope adapter camera attached to a standard dissection microscope (Leica S6D, Leica Microsystems GmbH). Each image included a standardized scalebar to allow for downstream measurements. Specimens were kept submerged in 1 cm of >95% ethanol while images were taken. To characterize the radulae of representative individuals, soft tissues were first separated from the shell and bisected latitudinally using a sharp scalpel blade. The anterior half of each specimen was then processed as follows: whole tissue was incubated in a 1.5 mL microcentrifuge tube containing a 10% solution of proteinase-k for 5–15 minutes at 56 °C. Incubation was monitored and terminated once tissue was visibly degraded, but not fully digested. The tissue was then removed from the heat source, pulse-vortexed 3 ×, and then rinsed into a clean glass petri dish using deionized (DI) water. Under a dissection scope, the radular ribbon was then extricated from any remaining soft tissue and removed to another clean glass petri dish containing DI water to further dilute the proteinase-k solution and prevent further breakdown of the radular ribbon. Silicon wafer chips cut into ~ 1 cm^3^ squares were used as mounting substrate for scanning electron microscopy (SEM). To mount the radula, a drop of DI water was placed onto a chip within which the radula was then placed and manipulated into a flat, teeth-up position using forceps or a sharp probe. The radula’s position was monitored and adjusted under a light microscope while the water was allowed to evaporate. Once dry, radulae naturally adhered to the chip’s surface and were then stored dry until imaging.

**Figure 2.**
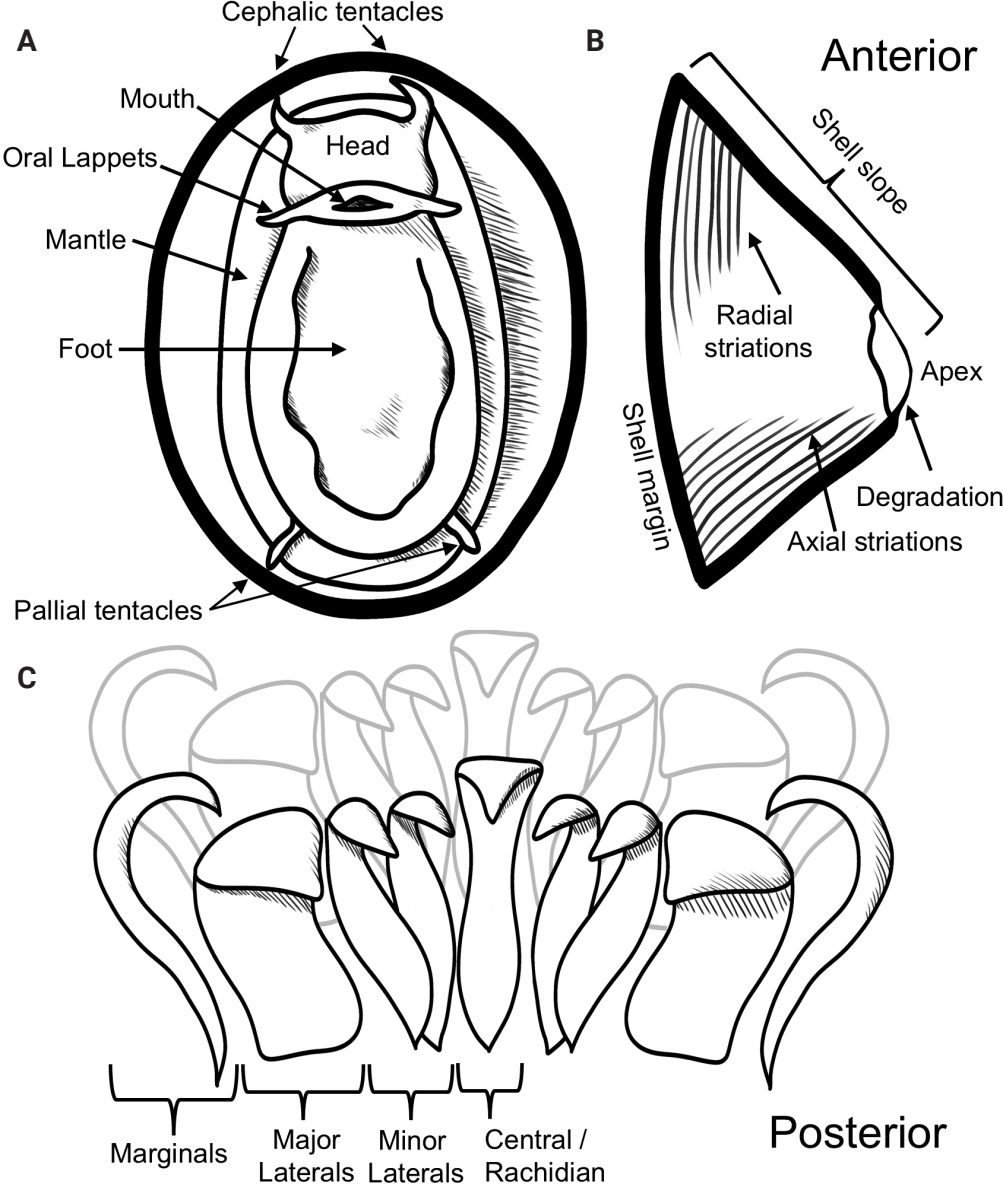
Selected character definitions for limpet **A** soft tissue **B** shells, and **C** radular teeth.

For specimens of *Bathyacmaea*, shell cross-sections were additionally imaged as shell microstructures are considered one of the few reliable morphological characters with which to identify species in this genus (Chen et al. 2019; Sato et al. 2020). Soft tissue was removed, then a cut was made with dissection scissors on one side of the shell from the shell margin to the apex (Fig. 2). Shell pieces cleaved apart naturally during this process along radial growth lines, and additional pieces were pried away using forceps. All pieces were sorted into more recently formed shell material (those closer to the shell margin) or older shell material (those closer to the apex). Shell pieces were then soaked in 0.5% commercial bleach (sodium hypochlorite) for 12 h. Pieces were then rinsed with de-ionized water, soaked in a 2% solution of 1 M hydrochloric acid for one minute, rinsed again, then left to dry before SEM imaging.

SEM was undertaken using a Quanta^TM^ 450 FEG scanning electron microscope (FEI 2012) in its high-vacuum setting at the Nano Instrumentation Center at Temple University’s College of Engineering. High-quality images were obtained without sputter coating. For imaging, *Bathyacmaea* shell pieces were adhered to a silicon wafer chip using carbon tape. Transverse (top-down) cross sections of the shell pieces were then imaged for both newer and older shell material.

### ﻿Genetic characterization

Representative individuals from each distinct shell morphotype were targeted for genetic sequencing. DNA was also extracted from the posterior half of each specimen that was bisected for radular isolation. Tissue was digested using a Qiagen Blood & Tissue DNA Extraction kit (QIAGEN, Valencia, CA). Extracts of DNA were obtained, quantitated using a Nanodrop 2000 spectrophotometer, and stored at -20 °C. A 710 base pair (bp) section of the mitochondrial cytochrome oxidase 1 (CO1) gene was targeted for sequencing using the primer pair LCO1490/HCO2198 (Folmer et al. 1994). A 275 bp section of the nuclear histone-3 (H3) gene was also targeted for sequencing using the primer pairs H3F/H3R or H3NF/H3NR (Colgan et al. 2000). Exact reaction conditions varied across polymerase chain reactions (PCR) (Suppl. material 1). Forward and reverse sequence reads were obtained from PCR products through GeneWiz (Azenta Life Sciences, South Plainfield, NJ), then cleaned and quality-assured using BioEdit (v. 7.2.5; Hall 1999). Reverse reads were reverse-complemented in MEGA-X (v. 10.2.6; Kumar et al. 2018) before all sequences were aligned using ClustalW (Thompson et al. 2003). One consensus sequence was then generated for each individual. Sequences were then run through NCBI’s nucleotide basic local alignment tool (BLAST) to find highly similar sequences. Sequences that yielded BLAST results within the same genus were grouped and investigated together.

Nesting of CRM specimens within a particular family and genus was assessed using published sequences from hypothesized sister species, genera, families, as well as representative sequences from unrelated gastropod subclasses (outgroups). For each phylogenetic investigation, novel and published sequences were aligned using ClustalW embedded within MEGA-X and the best-fit substitution model was determined using the MEGA-X model finder based on the lowest Bayesian Information Criterion. Maximum likelihood (ML) phylogenies were computed within MEGA-X using 10,000 bootstrap replicates. Bayesian phylogenies were computed within the joint programs BEAUTi and BEAST (v. 1.10.4; Suchard et al. 2018) using a strict time clock, yule speciation process (Gernhard 2008), and random starting tree in all cases. Unless otherwise stated, all other program settings were left as default. The maximum clade credibility tree was selected from the BEAST output using TreeAnnotator (v. 1.10.4) with 100,000 burn-in states. For both Bayesian and ML approaches, all base positions with less than 95% coverage were excluded from analyses. Resultant phylogenies were visualized using Fig. Tree (v. 1.4.4; Rambaut 2018) and finalized in Adobe Illustrator (v. 27.3.1). For the genus *Paralepetopsis*, which was particularly difficult to identify to species, we additionally employed the program Assemble Species by Automatic Partitioning (ASAP; Puillandre et al. 2021) to determine the number of likely species represented by our sequences. We also calculated average pairwise sequence distances between and within our sequences of *Paralepetopsis* using MEGA-X, a Jukes-Cantor substitution model, and the 95% pairwise deletion option.

## ﻿Results

### ﻿Morphological characterization

**Figure 3.**
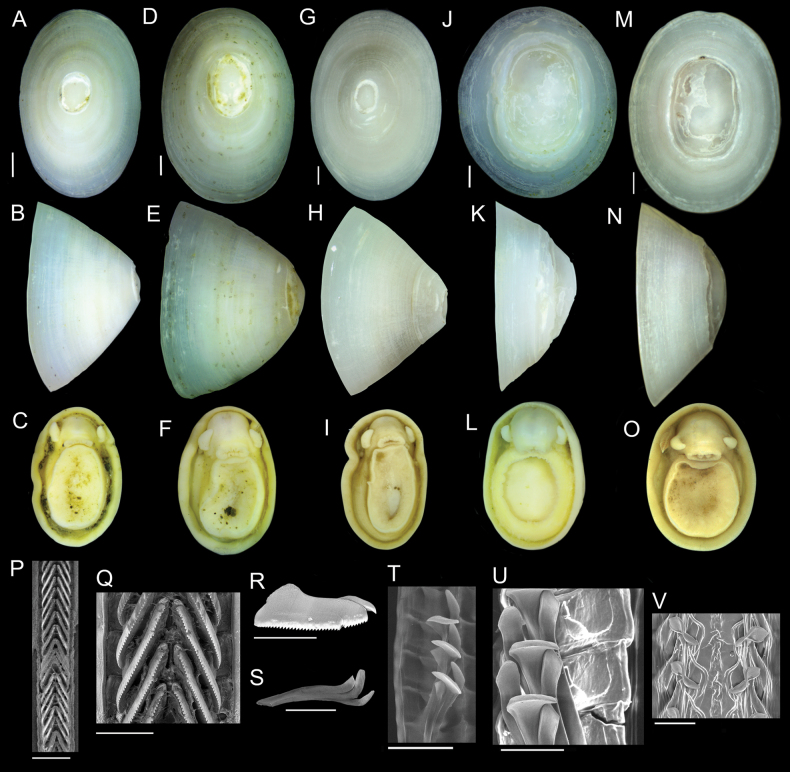
*Bathyacmaealevinae* sp. nov. **A–C** holotype from tubeworms at Jaco Scar, 1,724 m, AD4971, 17 October 2018 **D–F** paratype from tubeworms at Jaco Scar, 1,724 m, AD4971, 17 October 2018 **G–I** Sequenced specimen (GenBank Accession #OQ644578) from tubeworms at Jaco Scar, 1,760 m, AD4989, 4 November 2018 **J–L** paratype from mussels at Quepos Seep, 1,409 m, AD4924, 7 June 2017 **M–O** sequenced specimen (GenBank Accession #OQ644573) from mussels at Jaco Scar, 1,783 m, AD4977, 23 October 2018 **P** radula from specimen sampled from tubeworms **Q** closer view of the same radula **R** isolated radular tooth **S** isolated radular tooth from specimen sampled from mussels **T, U** radula from specimen sampled from mussels **V** under-developed (young) section of radula from the same specimen. Scale bars: 1 mm (**A–O**); 250 µm (**P**); 100 µm (**Q–T**); 50 µm (**U**);100 µm (**V**).

Nearly 4,000 limpets were collected from the Costa Rica Margin hydrocarbon seeps. All morphological characters are defined in Fig. 2. Two genera of patellogastropods were identified from the CRM: *Bathyacmaea* Okutani, Tsuchida & Fujikura, 1992 and *Paralepetopsis* McLean, 1990. Two morphotypes of *Bathyacmaea* were identified from the CRM: one inhabiting tubeworms (Fig. 3A–I), and one inhabiting mussel shells (Fig. 3J–O). Previous studies of *Bathyacmaea* have revealed substrate-dependent ecophenotypes within a single species (Chen et al. 2019), and it was thus considered a possibility that the two morphotypes identified here could constitute a single species. The specimens found on mussels most closely resembled the species *Bathyacmaeasubnipponica* Sasaki, 2003 and *Bathyacmaeanipponica* Okutani, Tsuchida & Fujikura, 1992, while those found on tubeworms most closely resembled *Bathyacmaeakanesunosensis* Sasaki, 2003. Early radular teeth of this species were found to be morphologically distinct from either of the mature morphotypes (Fig. 3V).

**Figure 4.**
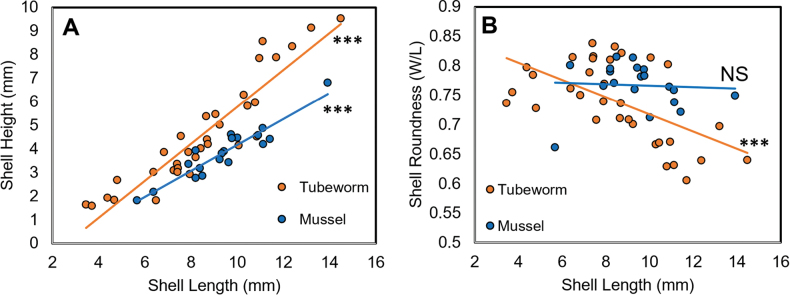
Divergent trends in growth among *Bathyacmaealevinae* sp. nov. Data from 52 individuals (Tubeworm, *n* = 33; Mussel, *n* = 19) are shown **A** specimens found on tubeworms become taller than those found on mussels as they grow, despite being similar in height at smaller sizes **B** specimens found on tubeworms become less round as they grow (*m* = -0.015, p < 0.001), while specimens found on mussels remain approximately the same roundness regardless of size (p > 0.1).

Measurements of *Bathyacmaea* specimens (*n* = 52) across the entire, sampled size range at the CRM found divergent trends in growth between substrates culminating in the morphological differences observed between the two substrates (Fig. 4). Specimens from tubeworms (*n* = 33) grew relatively taller than those found on mussels (*n* = 19) as shell length increased (Fig. 4A; tubeworm specimens: *m* = 0.78 p < 0.001, R2 = 0.85; mussel specimens: *m* = 0.55, p < 0.001, R2 = 0.87). Specimens from tubeworms also became significantly more oblique as shell length increased (Fig. 4B; *m* = -0.02, p < 0.001, R2 = 0.33), while those from mussel shells stayed around a constant measure of roundness (0.78), regardless of size (Fig. 4B; p > 0.1).

**Figure 5.**
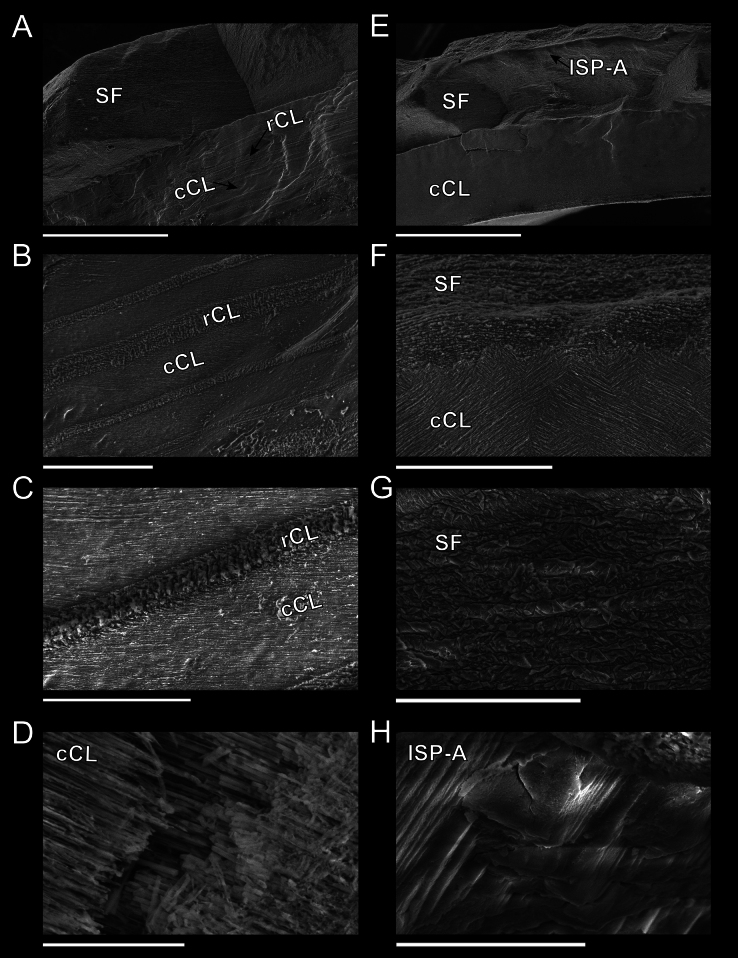
Shell microstructures of *Bathyacmaealevinae* sp. nov. from tubeworms at Jaco Scar, 1,785, October 2018. For all images, the outer shell is oriented to be at the top of the image, and the inner shell is at the bottom **A** cross section of newer shell (closer to the shell margin). Outermost layer shows semi-foliated structure, followed by alternating bands of crossed lamellar structure in concentric and radial orientations **B–D** close-up views of A **E** cross section of older shell (closer to the apex). Outermost layer shows irregular spherulitic prismatic type-A structure, followed by semi-foliated and concentric crossed lamellar structures **F–H** close-up views of E. Abbreviations: SF = semi-foliated structure, cCL = concentric crossed lamellar structure, rCL = radial crossed lamellar structure, ISP-A = spherulitic prismatic type-A structure. Scale bars: 300 µm (**A**); 50 µm (**B**); 20 µm (**C**); 10 µm (**D**); 300 µm (**E**); 40 µm (**F, G**); 5 µm (**H**).

Shell microstructures varied slightly between newer pieces of shell (Fig. 5A–C) and older pieces of shell (Fig. 5E–G). The outermost shell layers were formed of irregular spherulitic prismatic type-A (Fuchigami and Sasaki 2005; Sato et al. 2020), followed by semi-foliated structures, then by concentric crossed lamellar structures. In newer shell pieces, this concentric crossed lamellar structure was interspersed with bands of radial crossed lamellar structures (Fig. 5B,C), which were absent in older shell pieces (Fig. 5E, F).

**Figure 6.**
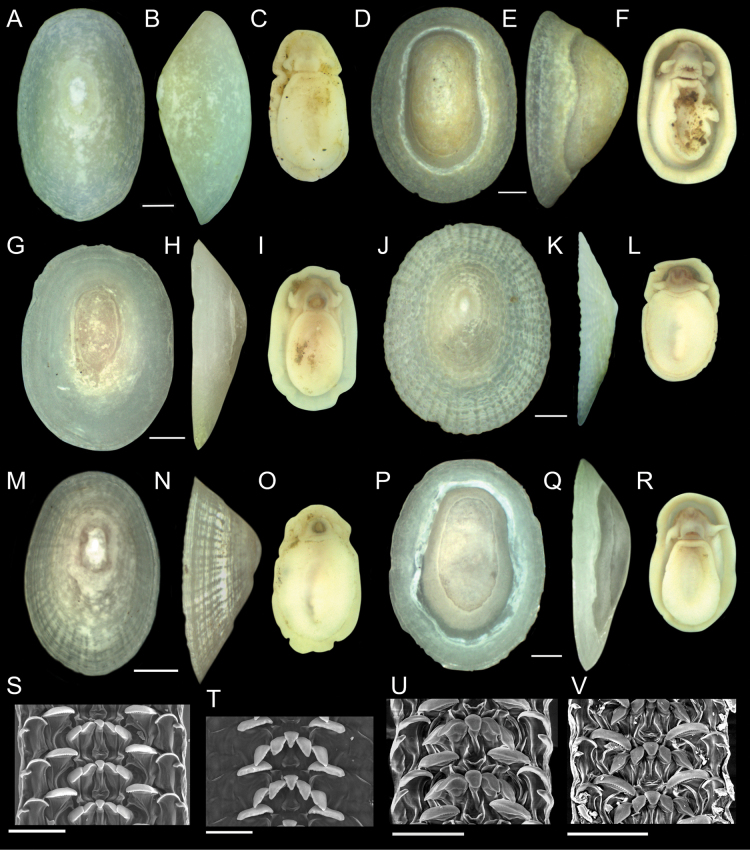
*Paralepetopsisvariabilis* sp. nov. **A–C** holotype from tubeworms at Mound 12, 995 m, AD4987, 2 November 2018 **D–F** sequenced clade 3 specimen (GenBank Accession #OQ644613) from plastic chip deployment, Jaco Scar, 1,796 m, AD4915, 17 October 2018 **G–I** sequenced clade 1 specimen (GenBank Accession #OQ644624) from mussels at Mound 12, 997 m, AD4978, 24 October 2018 **J–L** sequenced clade 1 specimen (GenBank Accession # OQ644614) from unknown substrate at Mound 12, 1,008 m, AD4501, 22 February 2009 **M–O** sequenced clade 2 specimen (GenBank Accession # OQ644619) from tubeworms, Jaco Scar, 1,724 m, AD4971, 17 October 2018 **P–R** sequenced clade 1 specimen (GenBank Accession # OQ644571) from mussels at Mound 12, 995 m, AD4985, 31 October 2018 **S–V** details of radular ribbons. Scale bars: 1 mm (**A–R**); 40 µm (**S**); 20 µm (**T**); 40 µm (**U**); 50 µm (**V**).

**Figure 7.**
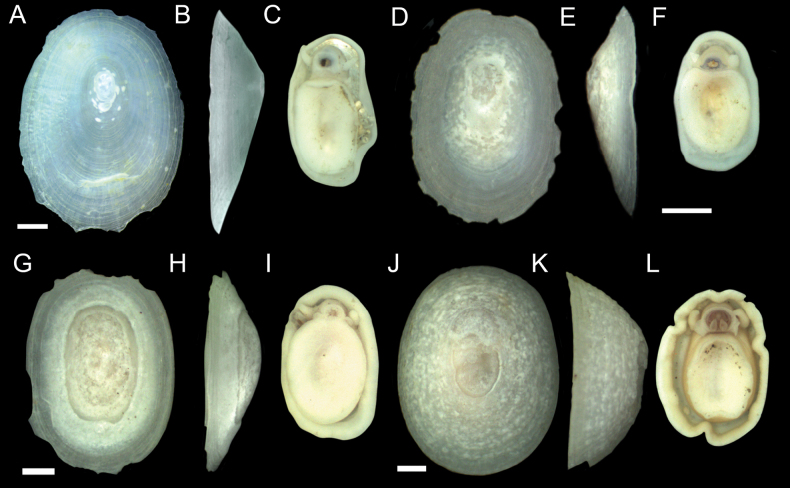
Specimens of *Paralepetopsis* representing clades 4 and 5 from Fig. 11**A–C** clade 4 specimen (GenBank Accession # OQ644570) found on tubeworms, Jaco Scar, 1,724 m, AD4971, 17 October 2018 **D–F** clade 4 specimen (GenBank Accession # OQ644599) found on mussels, Mound 12, 998 m, AD4908, 23 May 2017 **G–I** clade 5 specimen (GenBank Accession # OQ644577) from mussels, Jaco Scar, 1,783 m, AD4977, 23 October 2018 **J–L** clade 5 specimen (GenBank Accession # OQ644621) from plastic chip deployments, Jaco Scar, 1,796 m, AD4971, 17 October 2018. Radulae were unattainable for all specimens. Scale bars: 1 mm.

Specimens identified as *Paralepetopsis* encompassed an abundance of individuals and a wide variety of shell morphotypes, making sorting and identification difficult. All *Paralepetopsis* at the CRM exhibited white, semi-translucent shells and apexes that were consistently degraded and anteriorly offset (Figs 6, 7). Specimens were variable in many aspects of their morphology, including their shell sculpturing, the flatness of their shell margins, their shell slopes, and the shape and coloration of their soft tissues (Figs 6, 7). Some shells displayed axial sculpturing with minute beading (Fig. 6J, K), resembling their sister genus *Neolepetopsis* McLean, 1990. However, as later reported, genetic results consistently distinguished our specimens from members of *Neolepetopsis*. While radulae were not attained for all shell morphotypes identified (Fig. 7), radulae that were successfully examined showed consistency in their tooth shape and configuration (Fig. 6S–V). Subtle variations in morphology were also observed; The first major lateral teeth were found to be set at noticeably different angles depending on the individual and potentially the substrate. The erosion of the marginal teeth also varied between individuals, potentially due to differences in age or wear.

**Figure 8.**
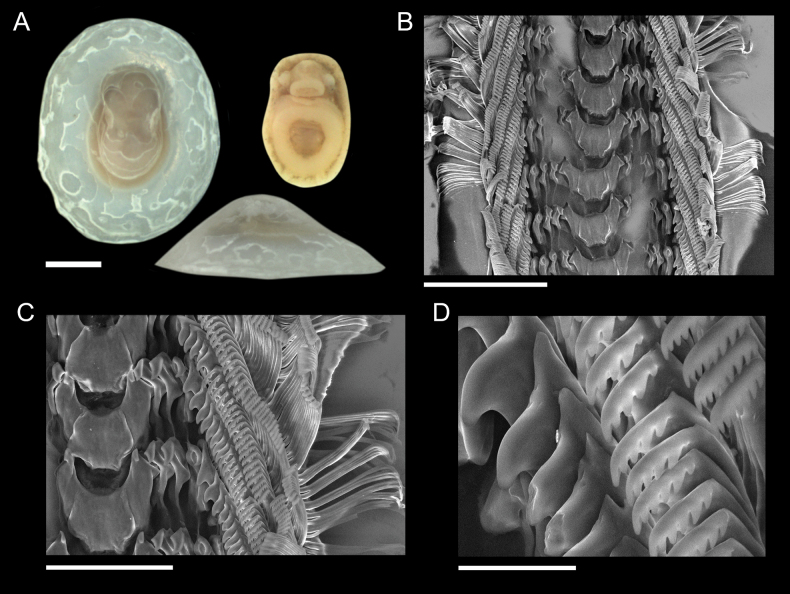
Specimen of *Pyropeltacorymba***A** sequenced specimen (GenBank Accession # OQ644631) found on mussel shells, Mound 12, 997 m, AD4978, 24 October 2018 **B–D** details of radula and major and minor lateral teeth. Scale bars: 1 mm (**A**); 100 µm (**B**); 50 µm (**C**); 10 µm (**D**).

Two genera of vetigastropods were identified from the CRM: *Pyropelta* McLean & Haszprunar, 1987 and *Lepetodrilus*McLean 1988. Specimens of *Pyropelta* were very small, with shells that were slightly ovate, semi-translucent, and that exhibited centrally located apexes of variable height (Fig. 8A). These specimens most closely resembled *P.corymba* McLean, 1992 or *P.musaica* McLean & Haszprunar, 1987, depending on their shell apex height. Soft tissue had a pinkish hue with two short, posterior epipodial tentacles and two cephalic tentacles (Fig. 8A). Radulae closely resembled those of a *P.corymba* specimen that was previously characterized from a Guaymas Basin hydrothermal vent (McLean 1992).

**Figure 9.**
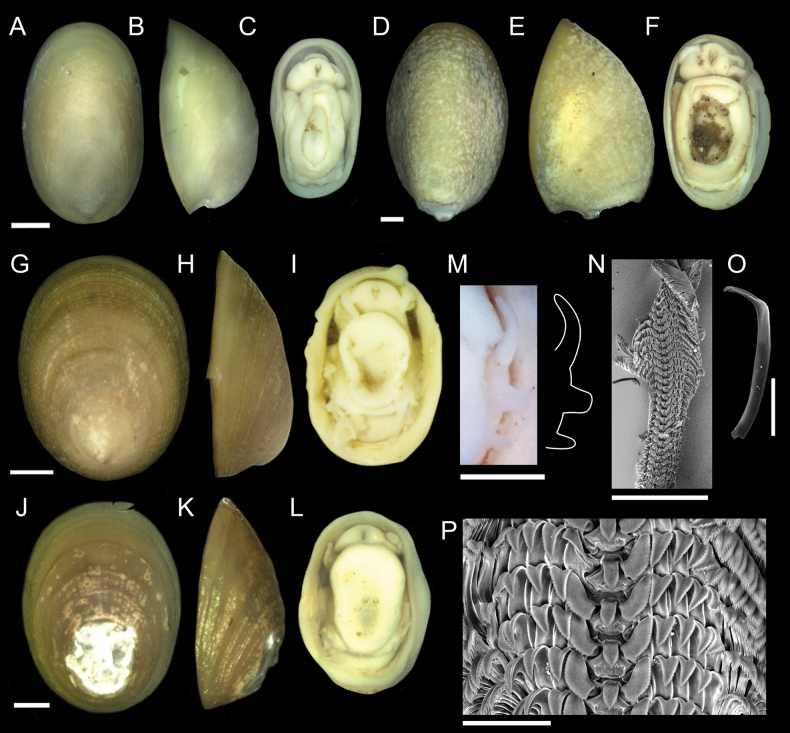
Specimens of *Lepetodrilusguaymasensis* and *Pseudolepetodriluscostaricensis* gen. et sp. nov. **A–C** sequenced *L.guaymasensis* specimen (GenBank Accession # OQ644591) from Mound 12, 998 m, AD4917, 1 June 2017 **D–F** additional *L.guaymasensis* specimen from Jaco Scar, 1,811 m, AD4912, 27 May 2017 **G–I***Pseudolepetodriluscostaricensis* sp. nov. holotype (**G–I**) and sequenced specimen (**J–L**) (GenBank Accession #OQ644586), both from tubeworms, Jaco Scar, 1,760 m, AD4989, 4 November 2018 **M** close up view of *P.costaricensis* epipodial tentacles, with tracing **N–P** radula morphology representative of this new genus **O** isolated marginal lateral tooth. Scale bars: 1 mm (**A–M**); 300 µm (**N**); 50 µm (**O**); 30 µm (**P**).

Morphological characterization of the vetigastropod genus *Lepetodrilus* yielded two distinct morphotypes. One morphotype matched the shell description of *L.guaymasensis* McLean, 1988 which displayed ovate apertures, variable shell heights, anterior narrowing of the shell and apexes which were very posteriorly shifted such that some overhung the posterior margin of the shell (Fig. 9A–F). The second morphotype had rounded apertures, low shell profiles, posteriorly shifted apexes that were more diminished than the preceding morphotype, three epipodial tentacles, and brown periostraca (Fig. 9G–M); These most closely resembled the species *L.shannonae* Warén & Bouchet, 2009. Radulae obtained from this second morphotype most closely resembled those of *L.guaymasensis* but had central and first lateral teeth that were distinct in shape (Fig. 9N, O).

**Figure 10.**
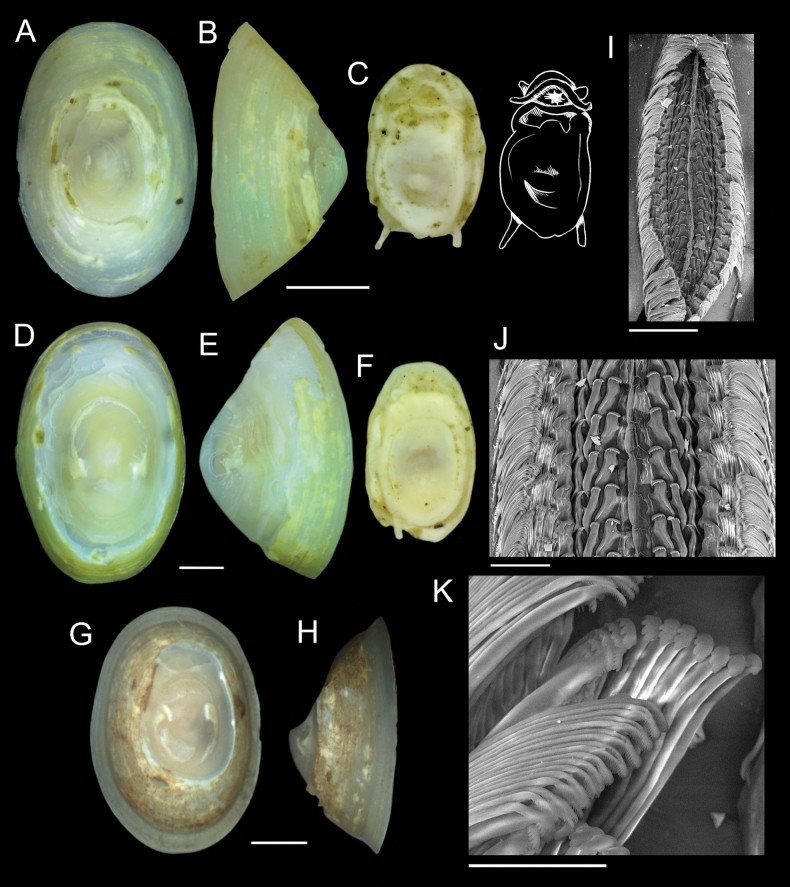
Specimens of *Cocculinamethana* sp. nov. **A, B** holotype from clam shells, Quepos Seep, 1,408 m, AD4924, 7 June 2017 **C** holotype soft tissue with tracing **D–F** paratype from tubeworms, Quepos Seep, 1064 m, AD4923, 7 June 2017 **G, H** sequenced specimen (GenBank Accession # OQ644629) from same location as previous showing intact periostracum prior to erosion with ethanol preservation. White dotted line denotes obstruction of image by forceps used to position the specimen **I–K** details of radula **K** details of marginal teeth. Scale bars: 1 mm (**A–H**); 150 µm (**I**); 50 µm (**J**); 20 µm (**K**).

Finally, one morphotype within the Neomphaliones genus *Cocculina* Dall, 1882 was identified. Specimens of *Cocculina* from the CRM had ovate apertures, moderately rounded shell margins, central shell apexes, and a golden-brownish periostracum (Fig. 10A, B, D, E). These shells most closely resembled those of *Cocculinajaponica* Dall, 1907. Notably, the periostracum of these specimens corroded significantly with prolonged ethanol preservation (Fig. 10G, H). Radulae of these specimens most closely resembled those of *C.cowani* McLean, 1987 but with distinct central teeth that form a narrow, defined ridge down the center of the radula (Fig. 10I, J).

### ﻿Genetic characterization

Individuals representing the full diversity of morphotypes collected were genetically barcoded for the mitochondrial cytochrome oxidase I (CO1) gene (*n* = 63) and the histone-3 (H3) gene (*n* = 19) (Table 1). All CO1 sequences generated were deposited in GenBank via NCBI under the accession numbers OQ644569–OQ644631. All H3 sequences generated were deposited under the accession numbers Q658576–OQ658595.

**Table 1.** Overview of gastropod limpet specimens collected from the Costa Rica Margin. Accession numbers refer to records in the NCBI Nucleotide Database (GenBank). Substrate abbreviations: B = Bone, C = Clams, M = Mussel, R = Rock, T = Tubeworm, W = Wood. Equipment dive number abbreviations: SD = Remotely operated vehicle SUBASTIAN dive, AD = human-operated vehicle Alvin dive. * = Locality not depicted on the region map in Fig. 1 (coordinates: 9.65, -85.88). ** = Cryptic species; only data from confirmed clade members are reported.

Genus	Total	Sites	Representative Sequence Accession Numbers			Depth	Substrate	Equipment dives
			Mound 12	Jaco Scar	Quepos Seep			
*Bathyacmaealevinae* sp. nov. (mussel) Holotype: SIO-BIC M22535	33	Jaco Scar	NA	CO1: OQ644573, OQ644574. H3: OQ658577.	NA	1780–1820	M, R	SD214, AD4914, AD4977
*Bathyacmaealevinae* sp. nov. (tubeworm) Holotype: SIO-BIC M22535	74	Jaco Scar	NA	CO1: OQ644578, OQ644584. H3: OQ658580.	NA	1720–1820	T	AD4911, AD4915, AD4971, AD4972, AD4989
*Cocculinamethana* sp. nov. Holotype: SIO-BIC M22533	64	Mound 12, Mound Jaguar*, Jaco Scar, Quepos Seep	NA	NA	CO1: OQ644628, OQ644629. H3: OQ658592, OQ658593	992–2000	T, W, B, C	SD230, AD4508, AD4913, AD4916, AD4924, AD4974
*Lepetodrilusguaymasensis*	765	Mound 12, Jaco Scar, The Thumb, Quepos Seep	CO1: OQ644589, OQ644591, OQ644592, OQ644593, OQ644594, OQ644595, OQ644596, OQ644602, OQ644603, OQ644604, OQ644605, OQ644611. H3: OQ658586, OQ658587	CO1: OQ644590, OQ644606. H3: OQ658585.	CO1: OQ644607, OQ644609, OQ644610.	990–1820	T, M, R	SD214, SD217, AD4511, AD4912, AD4915, AD4917, AD4922, AD4977, AD4984, AD4987
*Pseudolepetodriluscostaricensis* gen. et sp. nov. Holotype: SIO-BIC M22534	10	Jaco Scar	NA	CO1: OQ644586, OQ644587, OQ644588. H3: OQ658582, OQ658583, OQ658584.	NA	1760	T	AD4989
*Paralepetopsis* (all specimens)	>1420	Mound 12, Jaco Scar, The Thumb, Quepos Seep	See below	See below	See below	990–1820	T, M, R, C	AD4513, AD4908, AD4915, AD4916, AD4917, AD4922, AD4923, AD4971, AD4972, AD4977, AD4978, AD4984, AD4985, AD4987
*Paralepetopsisvariabilis* sp. nov. Clade 1** Holotype: SIO-BIC M22537	**	Mound 12, Jaco Scar	CO1: OQ644571, OQ644572, OQ644580, OQ644581, OQ644582, OQ644585, OQ644597, OQ644598, OQ644601, OQ644614, OQ644615, OQ644622, OQ644623, OQ644624. H3: OQ658589.	CO1: OQ644612, OQ644616, OQ644617.	NA	995–1741	T, M	AD4501, AD4908, AD4916, AD4922, AD4978, AD4984, AD4985, AD4987
*Paralepetopsisvariabilis* sp. nov. Clade 2** Holotype: SIO-BIC M22537	**	Mound 12, Jaco Scar	CO1: OQ644579, OQ644569, OQ644576.	CO1: OQ644618, OQ644620, OQ644619. H3: OQ658590.	NA	998–1796	T, M	AD4915, AD4922, AD4971
*Paralepetopsisvariabilis* sp. nov. Clade 3** Holotype: SIO-BIC M22537	**	Jaco Scar	NA	CO1: OQ644625, OQ644626, OQ644627, OQ644583, OQ644613, OQ644575. H3: OQ658578.	NA	1783–1796	T, M	AD4915, AD4971, AD4972, AD4977
*Paralepetopsis* sp. Clade 4**	**	Mound 12, Jaco Scar	CO1: OQ644599	CO1: OQ644570. H3: OQ658576.	NA	998–1724	T, M	AD4908, AD4971
*Paralepetopsis* sp. Clade 5**	**	Jaco Scar	NA	CO1: OQ644577, OQ644621. H3: OQ658579, OQ658591.	NA	1783–1796	M	AD4971, AD4977
*Pyropeltacorymba*	1692	Mound 12, The Thumb	CO1: OQ644600, OQ644608, OQ644630, OQ644631. H3: OQ658588, OQ658594, OQ658595	NA	NA	995–1080	T, M, R	SD217, AD4908, AD4917, AD4922, AD4978, AD4984

**Figure 11.**
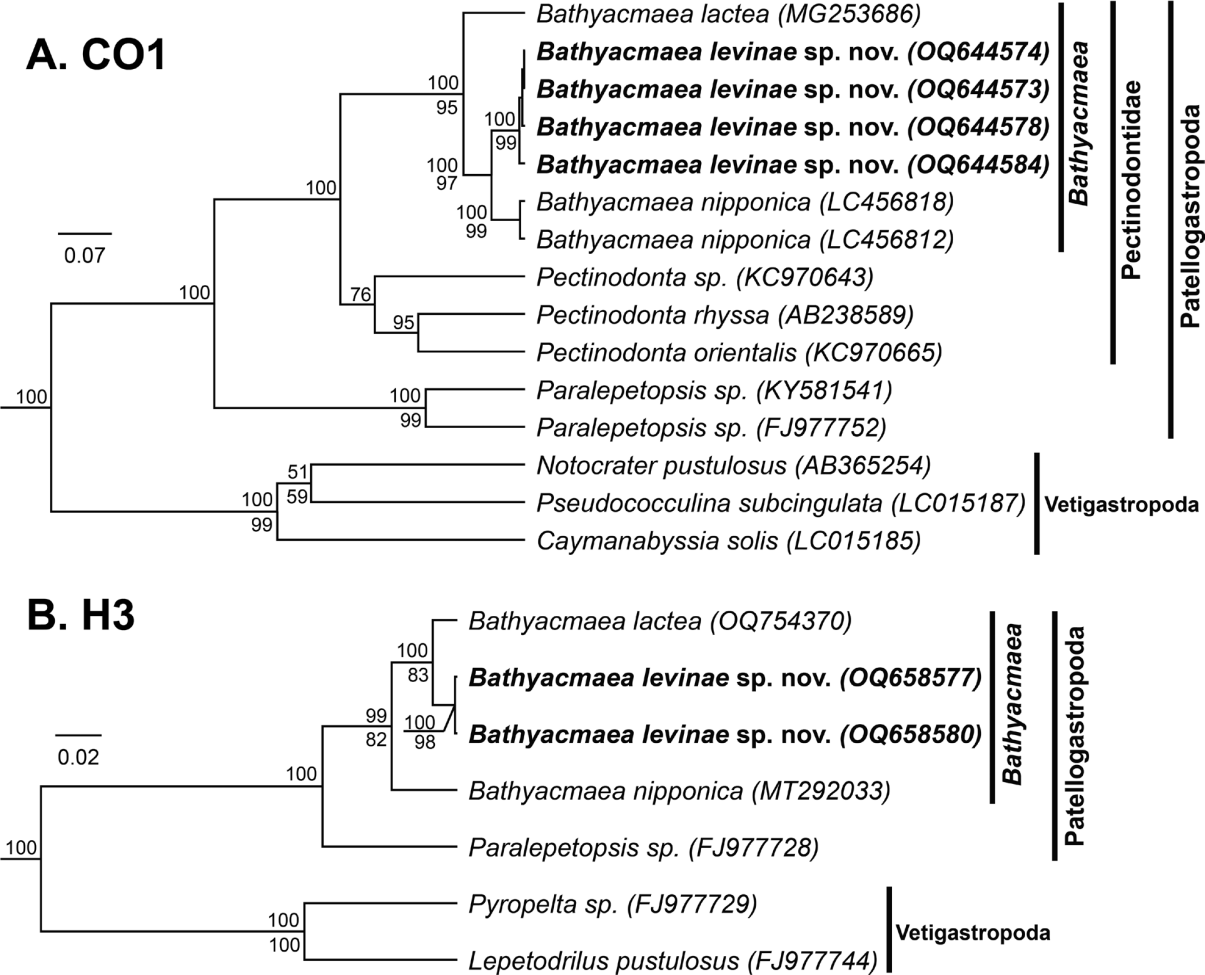
Bayesian phylogenies of *Bathyacmaea* and related genera **A** topology based on a 420-bp region of the mitochondrial CO1 gene and the HKY+G+I substitution model **B** topology based on a 258-bp region of the nuclear H3 gene and the GTR+G+I substitution model. Numbers above branch nodes represent Bayesian posterior probabilities. Numbers below branch nodes represent the proportion of replicate trees in which the associated taxa clustered together in the bootstrap test (10,000 replicates for CO1; 5,000 replicates for H3). Only values above 50 are shown. Novel sequences are bolded and highlighted. The trees are drawn to scale, with branch lengths representing the number of base substitutions accumulated over time.

From the Patellogastropods, specimens identified as *Bathyacmaea* Okutani, Tsuchida & Fujikura, 1992 were sequenced for CO1 (*n* = 4) and H3 (*n* = 2), evenly divided between mussel shell and tubeworm substrates (Table 1). Phylogenetic analyses supported their inclusion within the subclass Patellogastropoda (CO1: 100 (Bayesian Posterior Probability (BPP); H3: 100 (BPP)), the family Pectinodontidae (CO1: 100 (BPP)), and the genus *Bathyacmaea* (CO1: 100 (BPP), 95 (Bootstrap Likelihood (ML)); H3: 99 (BPP), 82 (ML)) (Fig. 11A, B). CO1 sequences from the tubeworm and mussel morphotypes showed minimal pairwise genetic divergence from one another (<2%). Our novel sequences were verified as being distinct from all other published *Bathyacmaea* sequences but were most similar to *B.nipponica* (GenBank Accession = MK341688; CO1 Percent Identity = 93.74%).

**Figure 12.**
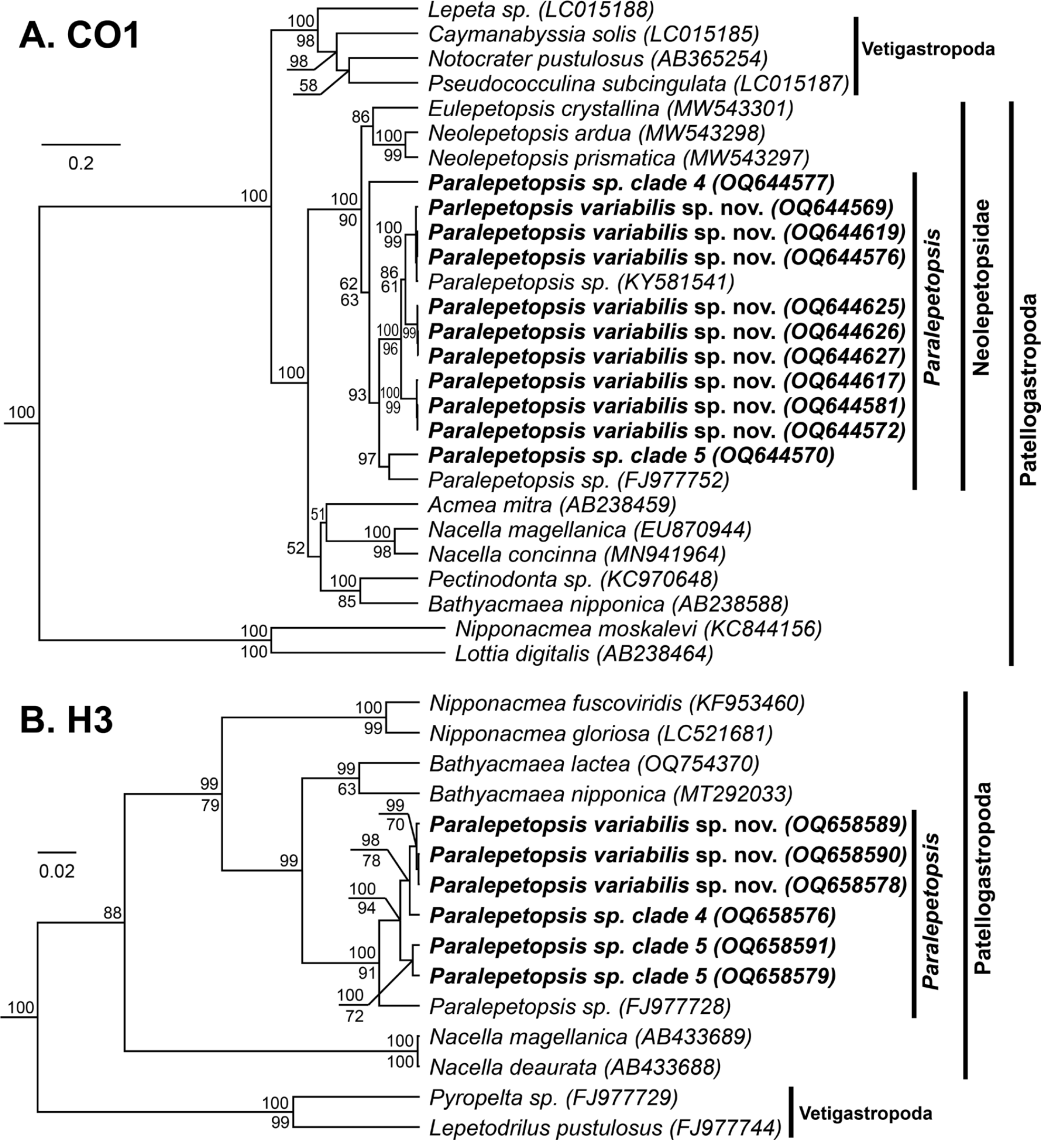
Bayesian phylogeny of *Paralepetopsis* and related genera **A** topology based on a 438-bp region of the mitochondrial CO1 gene and the GTR+G+I substitution model **B** topology based on a 247-bp region of the nuclear H3 gene and the GTR+G+I substitution model. Numbers above branch nodes represent Bayesian posterior probabilities. Numbers below branch nodes represent the proportion of replicate trees in which the associated taxa clustered together in the bootstrap test (10,000 replicates for CO1; 5,000 replicates for H3). Only values above 50 are shown. Novel sequences are bolded and highlighted. The tree is drawn to scale, with branch lengths representing the number of base substitutions accumulated over time.

Specimens identified as *Paralepetopsis* McLean, 1990 were sequenced for CO1 (*n* = 33) and H3 (*n* = 6; Table 1). Phylogenetic analyses supported these specimens’ inclusion within the subclass Patellogastropoda (CO1: 100 (BPP); H3: 100 (BPP)), the family Neolepetopsidae (CO1: 100 (BPP), 90 (ML)), and the genus *Paralepetopsis* (CO1: 100 (BPP), 90 (ML); H3: 100 (BPP), 91 (ML)) (Fig. 12A, B). Contrary to what was expected given their observed shell morphologies, no specimens grouped within the sister genus *Neolepetopsis* McLean, 1990. Our novel sequences were verified as being distinct from all other published *Paralepetopsis* sequences besides one (GenBank accession number KY581541; CO1 Percent Identity = 98%) obtained from an undescribed species from a Pescadero Basin hydrothermal vent.

**Figure 13.**
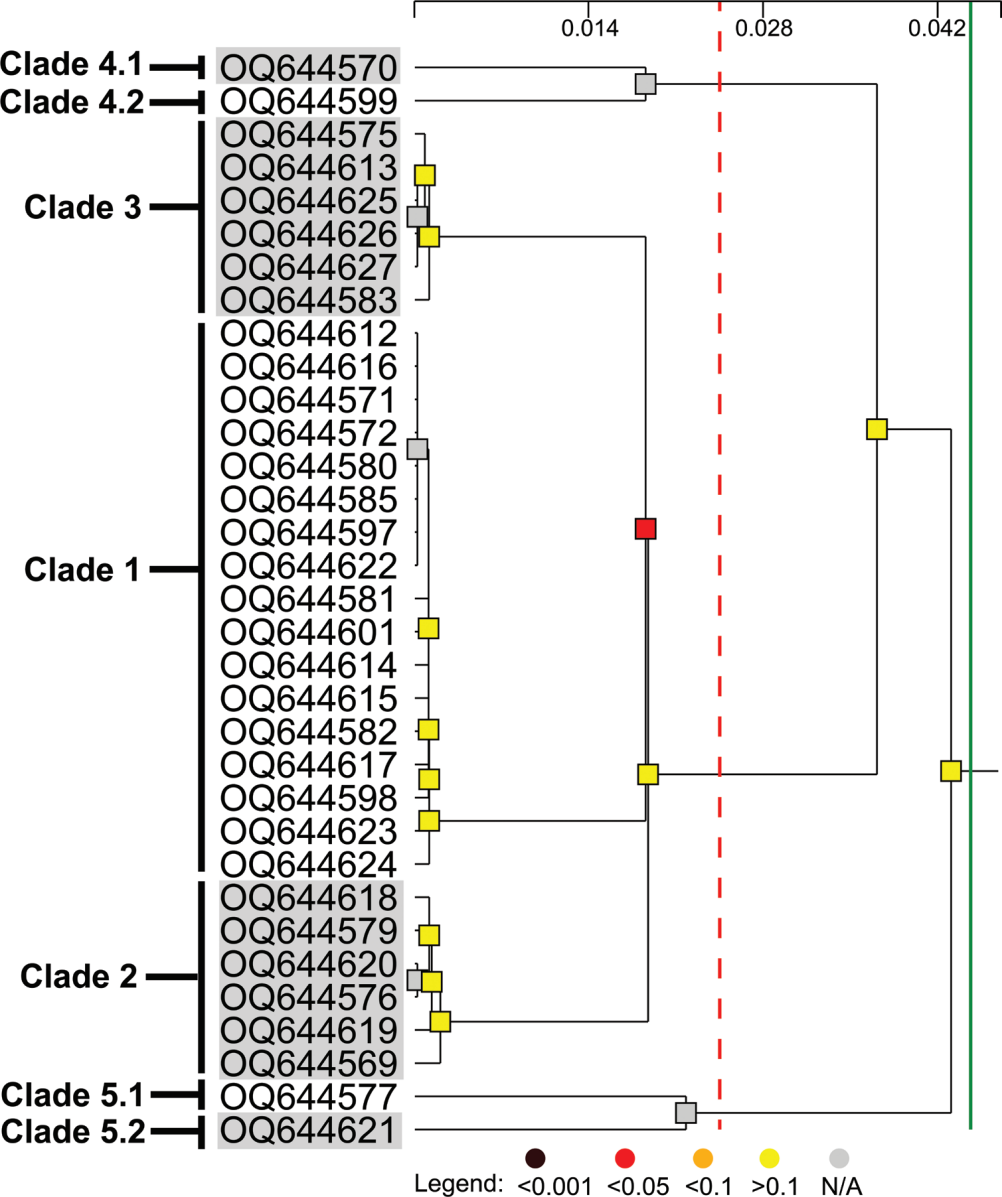
Automatic hierarchical partitioning of novel *Paralepetopsis*CO1 sequences. Dendrogram shown was generated by ASAP (Puillandre et al. 2021. The red dotted line denotes the distance threshold (Dt), and the solid green line denotes the grouping distance (Dc). Colored nodes represent the likelihood that each subset is distinct from a panmictic population. The scalebar at the top represents the relative barcode gap width (W).

It was difficult to discern the number of discrete species represented by our specimens of *Paralepetopsis*. To clarify this number, automatic hierarchical partitioning based on the mitochondrial CO1 gene was performed. Hierarchical clustering supported the existence of seven distinct subsets within our *Paralepetopsis* genetic dataset, with a threshold distance of 0.025 and a grouping distance of 0.043 (Fig. 13; p < 0.01). While this partitioning pattern was significant, two pairs of subsets could not be confidently distinguished from panmictic populations (Fig. 13; clades 4.1 and 4.2; clades 5.1 and 5.2; Puillandre et al. 2021). Notably, these four subsets were represented by only one sequence each. Thus, we collapsed these four subsets into two, conservatively designating five distinct clades from our results.

Pairwise sequence distances were then computed among these five conservative clades for both CO1 and H3 sequences. Sequence distances for CO1 fell between 5–13.6% and between 0–1.5% for H3 (Table 2). Clades 1–3 were not distinguishable based on H3 (0% for all pairs). For CO1, within-clade distances for clades 1–3 fell between 0.2–0.5% while between-clade distances fell between 5–7%. Within-clade distances for clades 4 and 5 were 4.6% and 5.4%, respectively. Thus, within-clade variation for clades 4 and 5 was comparable to the between-clade variation seen between clades 1, 2, and 3 (5% pairwise sequence divergence). These data dictate that one should either designate three distinct species (clades 1–3, 4, and 5) or designate seven distinct species (clades 1, 2, 3, 4.1, 4.2, 5.1, and 5.2). For the sake of being conservative in our delineations and given these clades’ morphological flexibility, overlapping distributions, and low H3 divergence, we opt for the former option and designate three distinct species among our *Paralepetopsis* specimens. Unfortunately, due to a shortage of representatives for clades 4 and 5, radulae and morphological variations were unable to be fully characterized, precluding formal description.

**Table 2.** The number of base substitutions per site from averaging over all sequences of *Paralepetopsis*. Analyses were conducted using the Jukes-Cantor substitution model, a gamma distribution rate of variation among sites, and pairwise deletion between sequence pairs. Cytochrome oxidase I distances between clades are given below the periphery (*n* = 32 sequences). Cytochrome oxidase I distances within clades are given at the periphery. Histone-3 distances between clades are given above the periphery (*n* = 6 sequences). Standard errors are given in parentheses and were estimated using 1,000 bootstrap replicates.

	Clade 1	Clade 2	Clade 3	Clade 4	Clade 5
**Clade 1**	0.003 (0.001)	0.000 (0.000)	0.000 (0.000)	0.006 (0.004)	0.015 (0.006)
**Clade 2**	0.067 (0.013)	0.005 (0.002)	0.000 (0.000)	0.006 (0.004)	0.015 (0.006)
**Clade 3**	0.050 (0.011)	0.050 (0.011)	0.002 (0.001)	0.006 (0.004)	0.015 (0.006)
**Clade 4**	0.107 (0.167)	0.111 (0.018)	0.108 (0.017)	0.046 (0.011)	0.015 (0.006)
**Clade 5**	0.120 (0.018)	0.136 (0.021)	0.127 (0.018)	0.121 (0.015)	0.054 (0.010)

**Figure 14.**
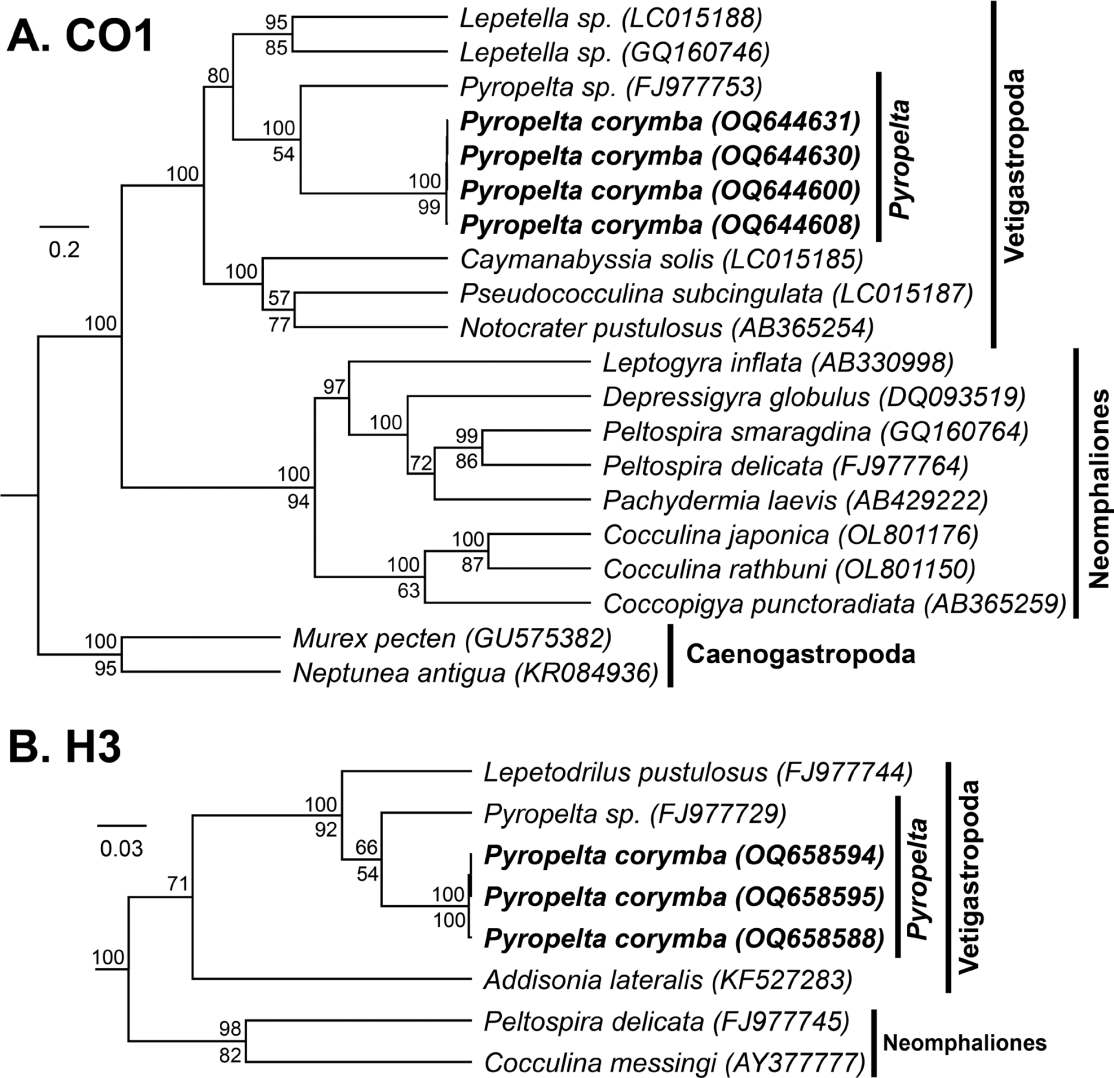
Bayesian phylogeny of *Pyropelta* and related genera **A** topology based on a 440-bp region of the mitochondrial CO1 gene and the GTR+G+I substitution model **B** topology based on a 321-bp region of the nuclear H3 gene and the GTR+G+I substitution model. Numbers above branch nodes represent Bayesian posterior probabilities. Numbers below branch nodes represent the proportion of replicate trees in which the associated taxa clustered together in the bootstrap test (10,000 replicates for CO1; 5,000 replicates for H3). Only values above 50 are shown. Novel sequences are bolded and highlighted. The tree is drawn to scale, with branch lengths representing the number of base substitutions accumulated over time.

For the vetigastropods, specimens identified as *Pyropelta* McLean & Haszprunar, 1987 were sequenced for CO1 (*n* = 4) and H3 (*n* = 3). These sequences supported these specimens’ inclusion within Vetigastropoda (CO1: 100 (BPP); H3: 100 (BPP)), and within the genus *Pyropelta* (CO1: 100 (BPP), 54 (ML); H3: 66 (BPP), 54 (ML)) (Fig. 14A, B). Genetic affinity to *P.corymba* and *P.musaica*, the species that ours most morphologically resembled, could not be assessed due to a lack of available sequences on GenBank. However, our sequences were nonetheless verified as being distinct from all other published *Pyropelta* sequences and were most similar to an unidentified species from the Gulf of Mexico (GenBank Accession = FJ977753; CO1 Percent Identity = 89.4%). Our novel CO1 sequences showed minimal pairwise genetic divergence from one another (< 1%).

**Figure 15.**
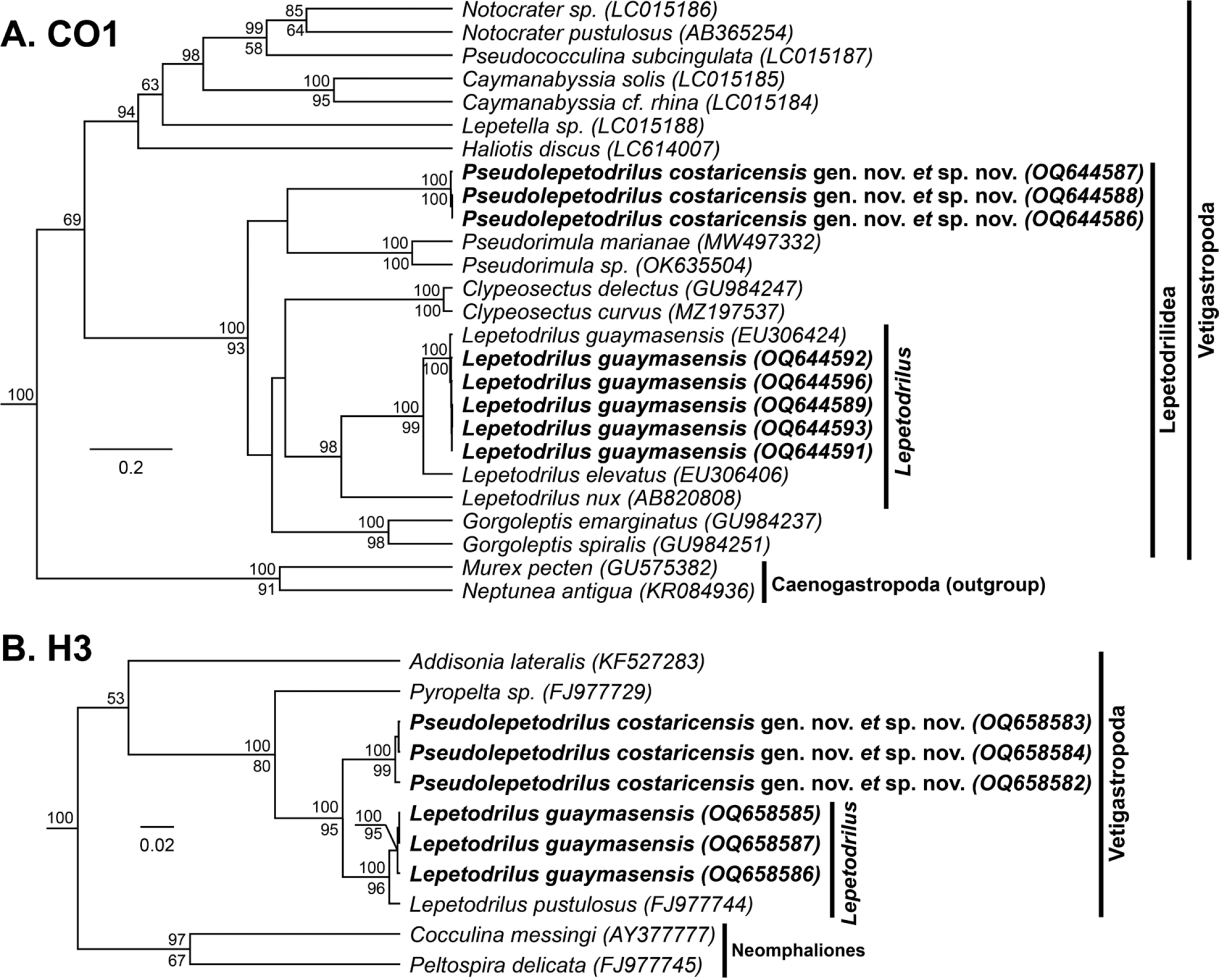
Bayesian phylogeny of *Lepetodrilus* and related genera **A** topology based on a 440-bp region of the mitochondrial CO1 gene and the GTR+G+I substitution model **B** topology based on a 308-bp region of the nuclear mitochondrial CO1 gene and the GTR+G+I substitution model. Numbers above branch nodes represent Bayesian posterior probabilities. Numbers below branch nodes represent the proportion of replicate trees in which the associated taxa clustered together in the bootstrap test (10,000 replicates for CO1; 5,000 replicates for H3). Only values above 50 are shown. Novel sequences are bolded. The tree is drawn to scale, with branch lengths representing the number of base substitutions accumulated over time.

The two morphotypes identified as *Lepetodrilus* McLean, 1988 were genetically characterized and supported as being within the superfamily Vetigastropoda (CO1: 100 (BPP); H3: 100 (BPP)) and the family Lepetodrilidae (CO1: 100 (BPP), 93 (ML); H3: 100 (BPP), 95 (ML)). One morphotype nested within the genus *Lepetodrilus* (CO1: 100 (BPP), 99 (ML); H3: 100 (BPP), 96 (ML)) and among the species *L.guaymasensis* with high confidence (CO1: 100 (BPP), 100 (ML)) (Fig. 15A, B). *Lepetodrilusguaymasensis* is a known species from the CRM matching the physical descriptions of our specimens, and thus this genetic affinity was expected (Johnson et al. 2008; GenBank accession number EU306419; CO1 Percent Identity = 100%). These novel CO1 sequences showed minimal pairwise genetic divergence from one another (< 1%).

The second morphotype, however, nested within the family Lepetodrilidae (CO1: 100 (BPP), 93 (ML); H3: 100 (BPP), 95 (ML)), but were excluded from the genus *Lepetodrilus* (CO1: 100 (BPP), 93 (ML); H3: 100 (BPP), 95 (ML)), despite morphological similarities. They were also excluded from all other Lepetodrilid genera (Fig. 14A, B). These sequences were verified as being distinct from all other published Lepetodrilidae sequences besides one unidentified specimen on GenBank (GenBank Accession number KJ566949; CO1 Percent Identity = 94.4%). These novel CO1 sequences showed minimal pairwise genetic divergence from one another (< 1%).

**Figure 16.**
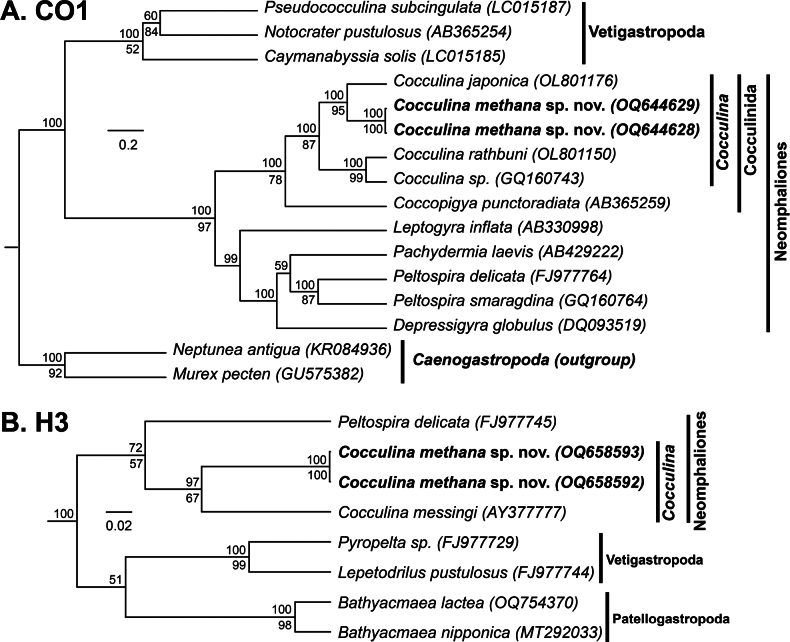
Bayesian phylogeny of *Cocculina* and related genera **A** topology based on a 436-bp region of the mitochondrial CO1 gene and the GTR+G+I substitution model **B** topology based on a 258-bp region of the nuclear H3 gene and the GTR+G+I substation model. Numbers above branch nodes represent Bayesian posterior probabilities. Numbers below branch nodes represent the proportion of replicate trees in which the associated taxa clustered together in the bootstrap test (10,000 replicates for CO1; 5,000 replicates for H3). Only values above 50 are shown. Novel sequences are bolded. The tree is drawn to scale, with branch lengths representing the number of base substitutions accumulated over time.

Finally, the Neomphaliones genus *Cocculina* Dall, 1882 was sequenced for CO1 (*n* = 2) and H3 (*n* = 2) (Table 1). Genetic characterization of these specimens supported their inclusion within the subclass Neomphaliones (CO1: 100 (BPP), 97 (ML); H3: 72 (BPP), 57 (ML)), the family Cocculinidae (CO1: 100 (BPP), 78 (ML)), and the genus *Cocculina* (CO1: 100 (BPP), 87 (ML); H3: 97 (BPP), 67 (ML)). (Fig. 16A, B). Pairwise distances between our CO1 sequences were found to be very low (< 2%). Our novel sequences were verified as being distinct from all other published *Cocculina* sequences but were most similar to *C.japonica* (GenBank accession number OL801181; CO1 Percent Identity = 88.4%).

### ﻿Institution codes

**SIO-BIC** Scripps Institute of Oceanography Benthic Invertebrate Collection

**MZUCR** Museo de ZoologÍa de la Universidad de Costa Rica

**EC** Erik Cordes, Personal Collection at Temple University

### ﻿New species and records

#### ﻿Subclass Patellogastropoda

**Family Pectinodontidae Pilsbry, 1891**

**Genus *Bathyacmaea* Okutani, Tsuchida & Fujikura, 1992**

##### ﻿ Bathyacmaea levinae sp. nov.

Taxon classificationAnimaliaPatellogastropodaPectinodontidae

2EE62F9E-B3B7-57F2-9090-E71BD64C8FA3

https://zoobank.org/3ACA68B0-D822-4518-BC96-F6345718B7D9

Fig. 3

###### Type material examined.

***Holotype*.** Costa Rica • whole organism; ethanol-fixed; Original label: “*Bathyacmaealevinae* holotype, 1, whole organism, AD4971, Costa Rica Margin, Jaco Scar, 9.11785, -84.8407, 1800 m, from tubeworms.”; SIO-BIC M22535. ***Paratypes***: • Same data as for holotype. Original label: “*Bathyacmaealevinae* paratype, 1, whole organism, AD4971, Costa Rica Margin, Jaco Scar, 9.11785, -84.8407, 1800m, from tubeworms.”. SIO-BIC M22536. Costa Rica • 2 specimens; same data as for holotype; Original label: “*Bathyacmaealevinae* paratype, 2, whole organisms, AD4971, Costa Rica Margin, Jaco Scar, 9.11785, -84.8407, 1720–1820 m, from tubeworms.”; MZUCR10674-01-02. Costa Rica • 2 specimens; Costa Rica Margin, Quepos Seep, 9.03174, -84.62158; hydrocarbon seep; mussels; 1,409 m; 7 June 2017; AT37-13 ALVIN Dive 4924 leg.; Paratype; whole organism; ethanol-fixed; Original label: “*Bathyacmaealevinae* paratype, 2, whole organisms, AD4924, Costa Rica Margin, Quepos Seep, 9.03174, -84.62158, 1409 m, from mussels.”; SIO-BIC M22532. Costa Rica • 2 specimens; Costa Rica Margin, Quepos Seep, 9.03174, -84.62158; hydrocarbon seep; mussels; 1,409 m; 7 June 2017; AT37-13 ALVIN Dive 4924 leg.; Paratype, whole organism; ethanol-fixed; Original label: “*Bathyacmaealevinae* paratype, 2, whole organisms, AD4924, Costa Rica Margin, Quepos Seep, 9.03174, -84.62158, 1409 m, from mussels.”; MZUCR10672-02-03.

###### Type locality.

Costa Rica • Costa Rica Margin, Jaco Scar, 9.11785, -84.8407; hydrocarbon seep; tubeworms; 1,720–1,820 m; 17 October 2018; AT42-03 ALVIN Dive 4971 leg.

###### Other material examined.

Costa Rica • 5 specimen(s); Costa Rica Margin, Jaco Scar; 9.117375, -84.8397; 1,811 m; 26 May 2017; AT37-13 ALVIN Dive 4911 leg.; Tubeworm, Erik Cordes Personal Collection (EC) 5739 • 5 specimen(s); Costa Rica Margin, Jaco Scar; 9.117375, -84.8397; 1,794 m; 29 May 2017; AT37-13 ALVIN Dive 4914 leg.; Mussel, EC5760 • 1 specimen(s); Costa Rica Margin, Jaco Scar; 9.11753, -84.83953; 1,886 m; 29 May 2017; AT37-13 ALVIN Dive 4914 leg.; Mussel, Scripps Benthic Invertebrate Collection (SIO-BIC) M16154 • 5 specimen(s); Costa Rica Margin, Jaco Scar; 9.117368, -84.839661; 1,796 m; 30 May 2017; AT37-13 ALVIN Dive 4915 leg.; Tubeworm, EC5815 • 10 specimen(s); Costa Rica Margin, Quepos Seep; 9.03048, -84.6202; 1,409 m; 7 June 2017; AT37-13 ALVIN Dive 4924 leg.; SIO-BIC M16201 • 10 specimen(s); Costa Rica Margin, Quepos Seep; 9.03048, -84.6202; 1,409 m; 7 June 2017; AT37-13 ALVIN Dive 4924 leg.; SIO-BIC M16179 • 10 specimen(s); Costa Rica Margin, Jaco Scar; 8.97043, -84.8429167; 1,724 m; 17 October 2018; AT42-03 ALVIN Dive 4971 leg.; Tubeworm, EC7745 • 10 specimen(s); Costa Rica Margin, Jaco Scar; 8.97043, -84.8429167; 1,724 m; 17 October 2018; AT42-03 ALVIN Dive 4971 leg.; Tubeworm, EC7420 • 10 specimen(s); Costa Rica Margin, Jaco Scar; 8.97043, -84.8429167; 1,724 m; 17 October 2018; AT42-03 ALVIN Dive 4971 leg.; Tubeworm, EC7419 • 1 specimen(s); Costa Rica Margin, Jaco Scar; 9.117433333, -84.83961667; 1,796 m; 17 October 2018; AT42-03 ALVIN Dive 4971 leg.; Tubeworm, SIO-BIC M16731 • 10 specimen(s); Costa Rica Margin, Jaco Scar; 8.97071, -84.8372817; 1,785 m; 18 October 2018; AT42-03 ALVIN Dive 4972 leg.; Tubeworm, EC7336 • 10 specimen(s); Costa Rica Margin, Jaco Scar; 8.97071, -84.8372817; 1,785 m; 18 October 2018; AT42-03 ALVIN Dive 4972 leg.; Tubeworm, EC7320 • 1 specimen(s); Costa Rica Margin, Jaco Scar; 9.11735, -84.83958333; 1,795 m; 18 October 2018; AT42-03 ALVIN Dive 4972 leg.; Tubeworm, SIO-BIC M16795 • 1 specimen(s); Costa Rica Margin, Jaco Scar; 9.11785, -84.83952833; 1,784 m; 19 October 2018; AT42-03 ALVIN Dive 4973 leg.; Tubeworm, SIO-BIC M16748 • 10 specimen(s); Costa Rica Margin, Jaco Scar; 8.97067, -84.839533; 1,783 m; 23 10 2018; AT42-03 ALVIN Dive 4977 leg.; Mussel, EC7548 • 11 specimen(s); Costa Rica Margin, Jaco Scar; 9.117567, -84.840718; 1,760 m; 4 November 2018; AT42-03 ALVIN Dive 4989 leg.; Tubeworm, EC8894 • 1 specimen(s); Costa Rica Margin, Jaco Scar; 9.117783333, -84.83945; 1,783 m; 4 November 2018; AT42-03 ALVIN Dive 4989 leg.; Rock, SIO-BIC M16943 • 8 specimen(s); Costa Rica Margin, Quepos Seep; 9.031816667, -84.62048333; 1,400 m; 5 November 2018; AT42-03 ALVIN Dive 4990 leg.; Mussel, SIO-BIC M17001 • 4 specimen(s); Costa Rica Margin, Quepos Seep; 9.031816667, -84.62048333; 1,400 m; 5 November 2018; AT42-03 ALVIN Dive 4990 leg.; Mussel, SIO-BIC M16988 • 2 specimen(s); Costa Rica Margin, Quepos Seep; 9.031816667, -84.62055; 1,401 m; 5 November 2018; AT42-03 ALVIN Dive 4990 leg.; Combined Slurp, SIO-BIC M16920 • 1 specimen(s); Costa Rica Margin, Jaco Scar; 9.1174, -84.839855; 1,803.1 m; 7 January 2019; FK19-0106 SUBASTIAN Dive 214 leg.; Rock, EC9345 • 1 specimen(s); Costa Rica Margin, Jaco Scar; 9.117775, -84.839525; 1,803 m; 7 January 2019; FK19-0106 SUBASTIAN Dive 214 leg.; Rock, EC9338 • 1 specimen(s); Costa Rica Margin, Jaco Scar; 9.1174, -84.839855; 1,803 m; 7 January 2019; FK19-0106 SUBASTIAN Dive 214 leg.; Rock, EC9337 • 1 specimen(s); Costa Rica Margin, Jaco Scar; 9.1174, -84.839855; 1,812.41 m; 7 January 2019; FK19-0106 SUBASTIAN Dive 214 leg.; Mussel, EC9323.

###### Diagnosis.

From tubeworms, *Bathyacmaealevinae* sp. nov. may be diagnosed by their flat, serrated radular teeth and high, conical shells lacking any obvious axial sculpturing. On mussels, *Bathyacmaealevinae* sp. nov. may be diagnosed through the combination of their ovate, evenly sloped, flattened shells lacking any obvious axial sculpturing with their radular characteristics. At the time of publication, these are the only *Bathyacmaea* species known from the Eastern Pacific Ocean.

###### Description.

***Shell*** (Figs 3A, B, 5): Specimens exhibit uncoiled, patelliform shells. Holotype measures 8.1 mm in length, 5.2 mm in width, and 4.7 mm in maximum height. Shell roundness (width ÷ length) is 0.65. Shell sculpturing and ornamentation lacking but fine radial growth lines are present. Very fine axial striations present but not raised. Aperture opening is ovate and aperture lip is thick and unornamented. Shell slope is flattened to mildly convex. Shell apex is degraded and centrally located. Protoconch is unknown. Shell is thick, white, and semi-translucent. Shell microstructures are (in order from the outermost shell layer to the innermost): irregular spherulitic prismatic type-A, semi-foliated, concentric crossed lamellar structures, and radial crossed lamellar structures (Fig. 5).

***Soft parts*** (Fig. 3C): Soft tissue is white-to-yellowish in color. Mantle is thick with a flat margin. Foot follows the shape of the shell aperture in terms of its roundness. Margin of the foot sole is flat. Pallial tentacles are lacking. Operculum is absent. Two cephalic tentacles are present which are short, thick, and placed low on the head. Bipectinate gill extends from behind animal’s right cephalic tentacle. Eyes are absent. Oral lappets are absent but the oral opening is lined with thickened tissue ornamented with very fine frilling.

***Radula*** (Fig. 3P–R): Radula was obtained from the sequenced specimen (Fig. 3G–I), whose shell measured 10 mm in length, 7.0 mm in width, and 6.8 mm in height. Docoglossate radula with formula 0+1+0+1+0. Radular ribbon measures ~ 240 µm across. Rachidian teeth highly diminished and obscured by laterals. Lateral teeth are long, robust, and consisting of three distinct cusps that appear to be fused together. Lateral teeth may measure up to 400 µm in length. The first, most anterior, cusp forms a single sharp hook lacking denticle. The second cusp is longer than the first and falls in line with the third cusp such that it creates one continuous ridge. Eight or nine short, sharp denticles are present on this second cusp. The third, most posterior, cusp is the longest (~ 3 × the length of the second cusp) and forms a robust, serrated ridge with 25 or more short, sharp denticles that are indistinguishable from those on the second cusp. The third cusp’s posterior end curves inward towards the radular ribbon. The connecting point of the lateral teeth to the radular ribbon is located near the posterior end of the third cusp. Marginal teeth lacking.

###### Variation.

Two distinct morphotypes of *Bathyacmaealevinae* sp. nov. are herein identified: One inhabiting tubeworms (Fig. 3A–I), and one inhabiting mussel shells (Fig. 3J–O). Holotype description applies to specimens found on tubeworms. *Bathyacmaealevinae* sp. nov. found on mussel shells differ in that they exhibit rounder apertures, flatter shell margins, lower shell profiles, and greater apex erosion (Fig. 3J–N). Paratype specimens from mussel shells measure between 7.9–11.0 mm in length, 6.4–9.0 mm in width, and 3.0–4.5 mm in height. Measures of shell roundness (width ÷ length) for these paratypes are all between 0.81–0.85, distinguishing them from the roundness of the holotype (0.65).

Radulae of specimens found on mussels also differ (Fig. 3S–U). Formula remains 0+1+0+1+0 with tricuspid laterals, reduced rachidian teeth, and no marginals. All cusps of the lateral teeth are located at the very anterior end of a long, thin tooth shaft which connects to the radular ribbon at its far posterior end (Fig. 3S). The first, most anterior cusp of the lateral teeth is similar between morphotypes, being sharp, hooked, and lacking denticles. The second cusp of specimens found on mussels resembles the first cusp in terms of thickness, but with a blunt outer edge that lacks denticles and points forward (perpendicular to the radular ribbon). The third, most posterior cusp is short, thick, lacking denticles, and is fused with the second cusp. This third cusp is truncated and forms a sharp barb which faces outwards when situated on the radular ribbon. Under-developed teeth of this species (Fig. 3V) further differ. Rachidian and minor lateral teeth are highly reduced; Reduced minor lateral teeth present as thin and strand-like with pointed anterior ends. Major laterals exhibit broad, un-serrated cusps whose outermost end is twisted backwards, forming a lemniscate shape. The first tooth cusp forms a sharp barb, similar to the mature radular tooth (Fig. 3S).

###### Distribution.

*Bathyacmaealevinae* sp. nov. has been collected from the hydrocarbon seep sites “Jaco Scar” (9.12, -84.84) and “Quepos Seep” (9.03, -84.62) at the Pacific Costa Rica Margin. This species was sampled from both mussels and tubeworms between 1,400–1,890 m depth.

###### Remarks.

Measurements of *Bathyacmaealevinae* sp. nov. across the entire, sampled size range at the CRM found divergent trends in growth between substrates culminating in the morphological differences observed (Fig. 4). These substrate-determined differences support previous studies that demonstrate radula and shell variability in *Bathyacmaea* (Chen et al. 2019). *Bathyacmaealevinae* sp. nov. found on mussels at the CRM most closely resemble the species *Bathyacmaeanipponica* Okutani, Tsuchida & Fujikura, 1992. However, *Bathyacmaealevinae* sp. nov. are genetically distinct from this species for both the mitochondrial CO1 gene and the nuclear histone-3 gene. Furthermore, the inner shell layers of *Bathyacmaealevinae* sp. nov. are comprised of concentric and radial crossed lamellar microstructures only, distinguishing them from *Bathyacmaeanipponica*, whose inner shell layers display an interspersion of semi-foliated microstructures and crossed lamellar structures (Sato et al. 2020). *Bathyacmaealevinae* sp. nov. from mussel shells also closely resemble *Bathyacmaeasubnipponica* Sasaki, 2003, but lack its cancellated shell sculpture. *Bathyacmaealevinae* sp. nov. found on tubeworms most closely resemble *Bathyacmaeakanesunosensis* Sasaki, 2003, though its distribution is highly distinct from our specimens. Due to a lack of sequences published for *B.kanesunosensis*, their genetic distinction remains unknown. At the time of publication, *Bathyacmaealevinae* sp. nov. is the only *Bathyacmaea* species found in the Eastern Pacific.

###### Etymology.

This species is named for Dr. Lisa A. Levin from Scripps Institute of Oceanography for her significant contribution to deep-sea knowledge, especially in regard to hydrocarbon seeps.

#### ﻿Family Neolepetopsidae McLean 1990

**Genus *Paralepetopsis*McLean 1990**

##### ﻿ Paralepetopsis variabilis sp. nov.

Taxon classificationAnimaliaPatellogastropodaNeolepetopsidae

6A7CCB6C-9F96-5625-9220-9EAB3AA0D7C8

https://zoobank.org/BD92F9DD-83FD-4B3D-9883-950DDD9D0454

Fig. 6

###### Type material examined.

***Holotype*.** Costa Rica • whole organism; ethanol-fixed; Original label: “Paralepetopsisvariabilis holotype, 1, whole organism, AD4987, Costa Rica Margin, Mound 12, 8.92982, -84.31167, 996 m, from tubeworms.”; SIO-BIC M22537. ***Paratypes***: Costa Rica • 9 specimens; same data as for holotype; Original label: “*Paralepetopsisvariabilis* paratype, 9, whole organisms, AD4987, Costa Rica Margin, Mound 12, 8.92982, -84.31167, 996 m, from tubeworms.”; SIO-BIC M22538. Costa Rica • 10 specimens; same data as for holotype; Original label: “*Paralepetopsisvariabilis* paratype, 10, whole organisms, AD4987, Costa Rica Margin, Mound 12, 8.9298, -84.31167, 996 m, from tubeworms.”; MZCR10675-01-10.

###### Type locality.

Costa Rica • Costa Rica Margin, Mound 12, 8.92982, -84.31167; hydrocarbon seep; tubeworms; 996 m; 2 November 2018; AT42-03 ALVIN Dive 4987 leg.

###### Other material examined.

Costa Rica • 11 specimen(s); Costa Rica Margin, Mound 11; 8.9208, -84.3054; 1,040 m; 25 February 2009; AT15-44 ALVIN Dive 4504 leg.; Tubeworm, SIO-BIC M11995 • 3 specimen(s); Costa Rica Margin, Jaco Scar; 9.1172, -84.8417; 1,866 m; 3 March 2009; AT15-44 ALVIN Dive 4509 leg.; SIO-BIC M12037 • 10 specimen(s); Costa Rica Margin, Mound 12; 8.9305, -84.3123; 1,001 m; 5 March 2009; AT15-44 ALVIN Dive 4511 leg.; SIO-BIC M12058 • 25 specimen(s); Costa Rica Margin, Mound 12; 8.93042, -84.31278; 999 m; 22 May 2017; AT37-13 ALVIN Dive 4907 leg.; SIO-BIC M16114 • 9 specimen(s); Costa Rica Margin, Jaco Scar; 9.11538, -84.83618; 1,859 m; 27 May 2017; AT37-13 ALVIN Dive 4912 leg.; SIO-BIC M16126 • 1 specimen(s); Costa Rica Margin, Jaco Scar; 9.11538, -84.83618; 1,859 m; 27 May 2017; AT37-13 ALVIN Dive 4912 leg.; SIO-BIC M16122 • 5 specimen(s); Costa Rica Margin, Jaco Scar; 9.117368, -84.839661; 1,796 m; 30 May 2017; AT37-13 ALVIN Dive 4915 leg.; Tubeworm, EC5815 • 3 specimen(s); Costa Rica Margin, Jaco Scar; 9.117368, -84.839661; 1,796 m; 30 May 2017; AT37-13 ALVIN Dive 4915 leg.; Tubeworm, EC5769 • 7 specimen(s); Costa Rica Margin, Jaco Scar; 9.117368, -84.839661; 1,796 m; 30 May 2017; AT37-13 ALVIN Dive 4915 leg.; Tubeworm, EC5731 • 49 specimen(s); Costa Rica Margin, Jaco Scar; 9.118023533, -84.84095552; 1,741 m; 31 May 2017; AT37-13 ALVIN Dive 4916 leg.; Tubeworm, EC5783 • 11 specimen(s); Costa Rica Margin, Jaco Scar; 9.1193, -84.84277; 1,854 m; 31 May 2017; AT37-13 ALVIN Dive 4916 leg.; SIO-BIC M16170 • 3 specimen(s); Costa Rica Margin, Mound 12; 8.930395, -84.3124245; 995 m; 1 June 2017; AT37-13 ALVIN Dive 4917 leg.; Tubeworm, EC5794 • 3 specimen(s); Costa Rica Margin, Mound 12; 8.9293, -84.315; 1,000 m; 1 June 2017; AT37-13 ALVIN Dive 4917 leg.; SIO-BIC M16161 • 81 specimen(s); Costa Rica Margin, Mound 12; 8.93046775, -84.31244503; 998 m; 5 June 2017; AT37-13 ALVIN Dive 4922 leg.; Mussel, EC5743 • 2 specimen(s); Costa Rica Margin, Quepos Seep; 9.03048, -84.6202; 1,409 m; 7 June 2017; AT37-13 ALVIN Dive 4924 leg.; SIO-BIC M16200 • 8 specimen(s); Costa Rica Margin, Quepos Seep; 9.03048, -84.6202; 1,409 m; 7 June 2017; AT37-13 ALVIN Dive 4924 leg.; SIO-BIC M16182 • 15 specimen(s); Costa Rica Margin, Quepos Seep; 9.03048, -84.6202; 1,409 m; 7 June 2017; AT37-13 ALVIN Dive 4924 leg.; SIO-BIC M16181 • 5 specimen(s); Costa Rica Margin, Jaco Scar; 8.97043, -84.8429167; 1,724 m; 17 October 2018; AT42-03 ALVIN Dive 4971 leg.; Tubeworm, EC7751 • 156 specimen(s); Costa Rica Margin, Jaco Scar; 8.97043, -84.8429167; 1,724 m; 17 October 2018; AT42-03 ALVIN Dive 4971 leg.; Tubeworm, EC7750 • 3 specimen(s); Costa Rica Margin, Jaco Scar; 8.97043, -84.8429167; 1,724 m; 17 October 2018; AT42-03 ALVIN Dive 4971 leg.; Tubeworm, EC7745 • 10 specimen(s); Costa Rica Margin, Jaco Scar; 8.97043, -84.8429167; 1,724 m; 17 October 2018; AT42-03 ALVIN Dive 4971 leg.; Tubeworm, EC7744 • 3 specimen(s); Costa Rica Margin, Jaco Scar; 8.97043, -84.8429167; 1,724 m; 17 October 2018; AT42-03 ALVIN Dive 4971 leg.; Tubeworm, EC10486 • 16 specimen(s); Costa Rica Margin, Jaco Scar; 8.97043, -84.8429167; 1,724 m; 17 October 2018; AT42-03 ALVIN Dive 4971 leg.; Tubeworm, EC10471 • 63 specimen(s); Costa Rica Margin, Jaco Scar; 9.117433333, -84.83961667; 1,796 m; 17 10 2018; AT42-03 ALVIN Dive 4971 leg.; SIO-BIC M16752 • 3 specimen(s); Costa Rica Margin, Jaco Scar; 9.117433333, -84.83961667; 1,796 m; 17 October 2018; AT42-03 ALVIN Dive 4971 leg.; Rock, SIO-BIC M16733 • 123 specimen(s); Costa Rica Margin, Jaco Scar; 8.97071, -84.8373; 1,785 m; 18 October 2018; AT42-03 ALVIN Dive 4972 leg.; Tubeworm, EC7346 • 6 specimen(s); Costa Rica Margin, Jaco Scar; 8.97071, -84.8373; 1,785 m; 18 10 2018; AT42-03 ALVIN Dive 4972 leg.; Tubeworm, EC7343 • 25 specimen(s); Costa Rica Margin, Jaco Scar; 9.11785, -84.83728; 1,785 m; 18 October 2018; AT42-03 ALVIN Dive 4972 leg.; Tubeworm, EC7340 • 1 specimen(s); Costa Rica Margin, Jaco Scar; 9.11735, -84.83958333; 1,795 m; 18 October 2018; AT42-03 ALVIN Dive 4972 leg.; SIO-BIC M16796 • 37 specimen(s); Costa Rica Margin, Jaco Scar; 9.1178, -88.839533; 1,783 m; 23 October 2018; AT42-03 ALVIN Dive 4977 leg.; Mussel, EC7556 • 1 specimen(s); Costa Rica Margin, Jaco Scar; 9.11775, -84.83953333; 1,783 m; 23 October 2018; AT42-03 ALVIN Dive 4977 leg.; SIO-BIC M16805 • 64 specimen(s); Costa Rica Margin, Mound 12; 8.9308, -84.31263; 997 m; 24 October 2018; AT42-03 ALVIN Dive 4978 leg.; Mussel, EC10473 • 37 specimen(s); Costa Rica Margin, Mound 12; 8.9308, -84.31263; 997 m; 24 October 2018; AT42-03 ALVIN Dive 4978 leg.; Mussel, EC10472 • 425 specimen(s); Costa Rica Margin, Mound 12; 8.9307, -84.3128; 997 m; 30 October 2018; AT42-03 ALVIN Dive 4984 leg.; Mussel, EC8314 • 30 specimen(s); Costa Rica Margin, Mound 12; 8.9307, -84.3128; 997 m; 30 October 2018; AT42-03 ALVIN Dive 4984 leg.; Mussel, EC10477 • 20 specimen(s); Costa Rica Margin, Mound 12; 8.9307, -84.3128; 997 m; 30 October 2018; AT42-03 ALVIN Dive 4984 leg.; Mussel, EC10476 • 6 specimen(s); Costa Rica Margin, Mound 12; 8.9299, -84.31299; 995 m; 31 October 2018; AT42-03 ALVIN Dive 4985 leg.; Mussel, EC10478 • 100 specimen(s); Costa Rica Margin, Mound 12; 8.92983, -84.31167; 995 m; 2 November 2018; AT42-03 ALVIN Dive 4987 leg.; Tubeworm, EC8615 • 1 specimen(s); Costa Rica Margin, Jaco Scar; 9.117783333, -84.83944667; 1,785 m; 4 November 2018; AT42-03 ALVIN Dive 4989 leg.; SIO-BIC M16974 • 2 specimen(s); Costa Rica Margin, Jaco Scar; 9.117783333, -84.83944667; 1,785 m; 4 November 2018; AT42-03 ALVIN Dive 4989 leg.; SIO-BIC M16973 • 2 specimen(s); Costa Rica Margin, Quepos Seep; 9.031816667, -84.62048333; 1,400 m; 5 November 2018; AT42-03 ALVIN Dive 4990 leg.; Mussel, SIO-BIC M16995 • 2 specimen(s); Costa Rica Margin, Quepos Seep; 9.031816667, -84.62048333; 1,400 m; 5 November 2018; AT42-03 ALVIN Dive 4990 leg.; Mussel, SIO-BIC M16994 • 3 specimen(s); Costa Rica Margin, Quepos Seep; 9.031816667, -84.62048333; 1,400 m; 5 November 2018; AT42-03 ALVIN Dive 4990 leg.; Mussel, SIO-BIC M16991 • 1 specimen(s); Costa Rica Margin, The Thumb; 9.1174, -84.839855; 1,074 m; 7 January 2019; FK19-0106 SUBASTIAN Dive 214 leg.; Mussel, EC9348 • 10 specimen(s); Costa Rica Margin, The Thumb; 9.1174, -84.839855; 1,074 m; 7 January 2019; FK19-0106 SUBASTIAN Dive 214 leg.; Mussel, EC9328 • 1 specimen(s); Costa Rica Margin, The Thumb; 9.1174, -84.839855; 1,074 m; 7 January 2019; FK19-0106 SUBASTIAN Dive 214 leg.; Mussel, EC9327 • 1 specimen(s); Costa Rica Margin, The Thumb; 9.05, -84.4; 1,074 m; 10 January 2019; FK19-0106 SUBASTIAN Dive 217 leg.; Tubeworm, EC9480 • 1 specimen(s); Costa Rica Margin, The Thumb; 9.05, -84.4; 1,074 m; 10 January 2019; FK19-0106 SUBASTIAN Dive 217 leg.; Mussel, EC9468 • 1 specimen(s); Costa Rica Margin, The Thumb; 9.05, -84.4; 1,074 m; 10 January 2019; FK19-0106 SUBASTIAN Dive 217 leg.; Mussel, EC9451 • 1 specimen(s); Costa Rica Margin, The Thumb; 9.05, -84.4; 1,074 m; 10 January 2019; FK19-0106 SUBASTIAN Dive 217 leg.; Mussel, EC9434.

###### Diagnosis.

*Paralepetopsisvariabilis* sp. nov. may be diagnosed by their ovate, white, semi-translucent shells showing fine, radial growth rings. This species also exhibits two cephalic tentacles which are short (they do not extend past the outer shell margin) and placed low on the head. Soft tissue is whiteish-yellow in color. However, the most reliable way to diagnose *Paralepetopsisvariabilis* sp. nov. is through DNA characterization, as morphology is highly variable within this species and intersects with other known species in the genus.

###### Description.

***Shell*** (Fig. 6A, B): Specimen exhibits uncoiled, patelliform shell. Holotype measures 6.8 mm in length, 4.1 mm in width, and 2.9 mm in maximum height. Shell roundness (width ÷ length) is ~ 0.61. Shell sculpturing and ornamentation lacking but fine radial growth lines are present. Aperture opening is ovate and aperture lip is thin and unornamented. Shell apex is degraded and anteriorly shifted. Anterior and posterior shell slopes are flattened to mildly convex. Shell is very thin, white, and semi-translucent.

***Soft parts*** (Fig. 6C): Soft tissue is white to yellowish in color. Mantle is thick with a mildly crumpled margin. Foot follows the shape of the shell aperture in terms of its roundness. Margin of the foot sole is flat. Pallial tentacles are lacking. Operculum is absent. Two cephalic tentacles are present which are short, thick, and placed low on the head. Eyes are absent. Very reduced oral lappets are present, as well as thickened tissue around the mouth ornamented with very fine frilling. The animal’s head has a slight brownish coloration and a high profile.

***Radula*** (Fig. 6S): Docoglossate radula with formula 2+1+2+1+2+1+2. Rachidian teeth have long shapes, with large triangular cusps at their anterior ends lacking serration. Rachidian teeth are flanked on either side by a pair of minor lateral teeth that are similar in shape to the rachidian. These two minor laterals also have triangular cusps lacking serrations and are slightly rotated inwards. The third (major) lateral tooth is distinct from the other two laterals, in that its cusp is very broad, flat, and perpendicular to the radular ribbon with 9–13 small serrations along its edge. These serrations become less pronounced near the tooth’s outer end. These major laterals are not in line with the others, being set slightly lower, approximately midway between the rows of rachidian and minor lateral teeth. There are two marginal teeth set on the outer edge and just below the major laterals. Marginals have very short, semi-lunate cusps that lack serrations. First outer marginals are ~ 2 × the size of the second outer marginals.

###### Variation.

*Paralepetopsisvariabilis* sp. nov. exhibits significant shell variation across specimens which makes distinguishing species based on morphology alone difficult. Shells may measure 5–10 mm with shell roundness varying between 0.6 and 0.8. While all specimens exhibit uncoiled, patelliform shells, specimens may exhibit axial sculpturing, radial sculpturing, both, or neither. Shell margins may vary in that they may be flat, convex, or rounded. Shell apexes were unanimously degraded and anteriorly shifted, but the degree of this erosion varies; Some shells have only the protoconch degraded, while others have the majority of their outer shell degraded. Anterior and posterior shell slopes may be flat or mildly rounded. Shells may be thickened, very thin, yellowish, white, or semi-translucent.

Radulae of this species are somewhat variable, with the third major lateral teeth being at noticeably different angles depending on the individual and, potentially, the substrate (Fig. 6S–V). The first major lateral teeth imaged from a specimen collected from tubeworms were perpendicular to the radular ribbon (Fig. 6S), those collected from plastic were comparatively rotated outwards (Fig. 6U), and those collected from mussels were comparatively rotated inwards (Fig. 6V). The presence or absence of marginal teeth also varies (Fig. 6T), which may be dependent on the amount of wear on the radula or the stage of radular tooth development. This hypothesis, however, requires further testing to validate.

The mantle and foot margins of specimens may vary from flat to crumpled. Coloration of soft tissues varies between specimens, with some exhibiting a distinct blue-to-purple pigmentation around the oral lappets, while others do not.

###### Distribution.

*Paralepetopsisvariabilis* sp. nov. has been collected from the hydrocarbon seep sites “Jaco Scar” (9.12, -84.84), “Quepos Seep” (9.03, -84.62), “Mound 11” (8.92, -84.31), and “Mound 12” (8.93, -84.31) from the Pacific Costa Rica Margin. This species was sampled from mussels, tubeworms, and rocks between 995–1,860 m depth. Specimens have also been found and genetically characterized from a Pescadero Basin hydrocarbon seep site (23.64, -108.39), collected by the ROV Tiburon during dive #756 from below 2000 meters depth.

###### Remarks.

*Paralepetopsisvariabilis* sp. nov. clade 1 shells resemble most closely those of *P.clementensis* (McLean 2008) but could be distinguished from them by having flat, rather than rounded, shell margins and flat, rather than convex, shell slopes. At least one specimen of clade 1 exhibited shell structuring that fits the description of the closely related genus *Neolepetopsis* (Fig. 6J, K; McLean 1990). However, such specimens occurring within the genus *Paralepetopsis* indicates that shell sculpturing may not be as taxonomically informative as previously thought. *Paralepetopsisvariabilis* sp. nov. clade 2 specimens most closely resembled *P.clementensis* (McLean 2008); however, the axial striations exhibited by these individuals are distinct and are instead most like *P.tunnicliffae* (McLean 2008). Clade 2 specimens may be distinguished from *P.tunnicliffae* in that their shell margins are rounded, rather than flat. Radulae obtained from *Paralepetopsis* clade 2 appears most like *P.tunnicliffae* in their reduced lateral marginal teeth. *Paralepetopsisvariabilis* sp. nov. clade 3 specimens most closely resembled *P.clementensis* (McLean 2008); however, they lack the convex shell slopes typical of this species. Radulae of this species, overall, resembled those of *P.ferrugivora*, but have a distinct major lateral tooth shape (Warén and Bouchet 2001).

###### Etymology.

The species name *variabilis* is Latin for variable, referring to the notable and confounding shell variation observed in this species.

#### ﻿Subclass Vetigastropoda

**Family Pyropeltidae McLean & Haszprunar, 1987**

**Genus *Pyropelta* McLean & Haszprunar, 1987**

##### ﻿ Pyropelta corymba

Taxon classificationAnimaliaVetigastropodaPyropeltidae

McLean & Haszprunar, 1987

7269015F-784A-59C0-BEA9-7186D71ECFBF

Fig. 8

###### New records.

Costa Rica • 13 specimen(s); Costa Rica Margin, Mound 12; 8.93075, -84.31252; 998 m; 23 May 2017; AT37-13 ALVIN Dive 4908 leg.; Mussel, 4908_MP_12 • 4 specimen(s); Costa Rica Margin, Mound 12; 8.930395, -84.3124245; 995 m; 1 June 2017; AT37-13 ALVIN Dive 4917 leg.; Rock, EC5803 • 213 specimen(s); Costa Rica Margin, Mound 12; 8.93046775, -84.31244503; 998 m; 5 June 2017; AT37-13 ALVIN Dive 4922 leg.; Mussel, EC5741 • 973 specimen(s); Costa Rica Margin, Mound 12; 8.9308, -84.31263; 997 m; 24 October 2018; AT42-03 ALVIN Dive 4978 leg.; Mussel, EC7743 • 425 specimen(s); Costa Rica Margin, Mound 12; 8.9307, -84.3128; 997 m; 30 October 2018; AT42-03 ALVIN Dive 4984 leg.; Mussel, EC8314 • 7 specimen(s); Costa Rica Margin, Mound 12; 8.9307, -84.3128; 997 m; 30 October 2018; AT42-03 ALVIN Dive 4984 leg.; Tubeworm, EC10475 • 1 specimen(s); Costa Rica Margin, The Thumb; 9.05, -84.4; 1,072 m; 10 January 2019; FK19-0106 SUBASTIAN Dive 217 leg.; Mussel, EC9480 • 1 specimen(s); Costa Rica Margin, The Thumb; 9.05, -84.4; 1,072 m; 10 January 2019; FK19-0106 SUBASTIAN Dive 217 leg.; Mussel, EC9451 • 1 specimen(s); Costa Rica Margin, The Thumb; 9.05, -84.4; 1,072 m; 10 January 2019; FK19-0106 SUBASTIAN Dive 217 leg.; Mussel, EC9468 • 1 specimen(s); Costa Rica Margin, The Thumb; 9.05, -84.4; 1,072 m; 10 January 2019; FK19-0106 SUBASTIAN Dive 217 leg.; Mussel, EC9434.

###### Remarks.

Specimens of *Pyropeltacorymba* are herein confirmed from the hydrocarbon seep sites “Jaco Scar” (9.12, -84.84), “Mound 12” (8.93, -84.31), and “The Thumb” (9.05, -84.39) from the Pacific Costa Rica Margin. This species was sampled from mussels, tubeworms, rocks, and wood between 995–1,887 m depth. This extends the known range of this species southward from the Californian coast and Gulf of California.

#### ﻿Family Lepetodrilidae McLean, 1988

**Genus *Lepetodrilus* McLean, 1988**

##### ﻿ Lepetodrilus guaymasensis

Taxon classificationAnimaliaVetigastropodaLepetodrilidae

McLean, 1988

382DF24A-71EA-5C34-82B6-5CBD31C5BA5D

Fig. 9

###### New records.

Costa Rica • 13 specimen(s); Costa Rica Margin, Jaco Scar; 9.117323, -84.839671; 1,795 m; 27 May 2017; AT37-13 ALVIN Dive 4912 leg.; Tubeworm, EC5737 • 3 specimen(s); Costa Rica Margin, Jaco Scar; 9.117368, -84.839662; 1,796 m; 30 May 2017; AT37-13 ALVIN Dive 4915 leg.; Tubeworm, EC5811 • 10 specimen(s); Costa Rica Margin, Mound 12; 8.930395, -84.3124245; 995 m; 1 June 2017; AT37-13 ALVIN Dive 4917 leg.; Tubeworm, EC5798 • 48 specimen(s); Costa Rica Margin, Mound 12; 8.93046775, 84.31244503; 998 m; 5 June 2017; AT37-13 ALVIN Dive 4922 leg.; Mussel, EC5746 • 2 specimen(s); Costa Rica Margin, Jaco Scar; 8.97067, -84.839533; 1,783 m; 23 October 2018; AT42-03 ALVIN Dive 4977 leg.; Mussel, EC7553 • 247 specimen(s); Costa Rica Margin, Mound 12; 8.9307, -84.3128183; 997 m; 30 October 2018; AT42-03 ALVIN Dive 4984 leg.; Mussel, EC8313 • 5 specimen(s); Costa Rica Margin, Mound 12; 8.92983, -84.3131; 995 m; 2 November 2018; AT42-03 ALVIN Dive 4987 leg.; Tubeworm, EC8562 • 1 specimen(s); Costa Rica Margin, Jaco Scar; 9.117775, -84.839525; 1,803 m; 7 January 2019; FK19-0106 SUBASTIAN Dive 214 leg.; Rock, EC9336 • 319 specimen(s); Costa Rica Margin, Jaco Scar; 9.11741, -84.839632; 1,812.41 m; 7 January 2019; FK19-0106 SUBASTIAN Dive 214 leg.; Mussel, EC9330 • 2 specimen(s); Costa Rica Margin, The Thumb; 9.048849447, -84.39383397; 1,071.5 m; 10 January 2019; FK19-0106 SUBASTIAN Dive 217 leg.; Mussel, EC9500 • 1 specimen(s); Costa Rica Margin, The Thumb; 9.048835323, -84.39417277; 1,075 m; 10 January 2019; FK19-0106 SUBASTIAN Dive 217 leg.; Mussel, EC9488 • 1 specimen(s); Costa Rica Margin, The Thumb; 9.048821, -84.394156; 1,074 m; 10 January 2019; FK19-0106 SUBASTIAN Dive 217 leg.; Tubeworm, EC9480 • 1 specimen(s); Costa Rica Margin, The Thumb; 9.048871, -84.393744; 1,073 m; 10 January 2019; FK19-0106 SUBASTIAN Dive 217 leg.; Mussel, EC9468 • 1 specimen(s); Costa Rica Margin, The Thumb; 9.048836, -84.393773; 1,072 m; 10 January 2019; FK19-0106 SUBASTIAN Dive 217 leg.; Mussel, EC9451 • 1 specimen(s); Costa Rica Margin, The Thumb; 9.048866, -84.394112; 1,073 m; 10 January 2019; FK19-0106 SUBASTIAN Dive 217 leg.; Mussel, EC9434.

###### Remarks.

Specimens of *Lepetodrilusguaymasensis* are herein confirmed from the hydrocarbon seep sites “Jaco Scar” (9.12, -84.84), “Quepos Seep” (9.03, -84.62), “Mound 12” (8.93, -84.31), and “The Thumb” (9.05, -84.39) from the Pacific Costa Rica Margin. This species was sampled from mussels, tubeworms, and rocks between 995–1,800 m depth.

##### ﻿ Pseudolepetodrilus gen. nov.

Taxon classificationAnimaliaVetigastropodaLepetodrilidae

A2E73FCA-ACEB-5D09-8EE7-2537F43F187D

https://zoobank.org/CD35F6A5-6AAA-45CF-9D10-1B38CA8A20D6

Fig. 9

###### Type species.

*Pseudolepetodriluscostaricensis* sp. nov.

###### Diagnosis.

*Pseudolepetodrilus* gen. nov. have a complete shell with fine radial and concentric sculptures, penis originating at the right side of the head, and three pairs of posterior epipodial tentacles.

###### Description.

***Shell*** (Fig. 9G, H): Specimens exhibit patelliform shells with moderate elevation. Apex of shell is located at the posterior end of the shell. Fine concentric radial sculpturing and axial sculpturing present. The aperture and shell margin are ovate and unornamented. Shell is robust with a thick, greenish brown periostracum covering the outer shell and wrapping over the aperture lip.

***Soft parts*** (Fig. 9I, M): One pair of short cephalic tentacles are located on the head. One pair of epipodial tentacles are located approximately midway down the foot, with one tentacle present on either side of the organism. Three pairs of epipodial tentacles are present at the posterior end of the organism. These posterior tentacles are short and thin; They do not extend past the shell margin. A thick, triangular penis extends from beneath the right cephalic tentacle. Mouth is V-shaped. Oral lappets are lacking.

***Radula*** (Fig. 9N, O): Radula is rhipidoglossate in configuration and is symmetrical. Rachidian tooth is sharp and triangular, lacking denticles. One broad, major lateral tooth on either side of the rachidian flanked by four minor lateral teeth all with triangular cusps: Numerous (15+) marginal teeth flank the minor lateral teeth on either side, each exhibiting spatulate cusps with short denticles.

###### Remarks.

*Pseudolepetodrilus* gen. nov. have a complete shell, penis originating at the right side of the head, and three pairs of posterior epipodial tentacles. *Lepetodrilus* have a complete shell, penis originating at the right side of the head, and two pairs of posterior epipodial tentacles. *Gorgoleptis* have a complete shell, penis originating from the left side of the head, and two pairs of posterior epipodial tentacles. *Clypeosectus* McLean, 1989 has a slit shell and three pairs of posterior epipodial tentacles. *Pseudorimula* McLean, 1989 has a slit shell and four pairs of posterior epipodial tentacles.

The radulae of this new genus most closely resembles that of *Lepetodrilus* in that they both have a broad, oblique, first major lateral followed by laterals that rise to a peak at the third tooth and then descend away from the short, triangular rachidian. However, while the major laterals of *Lepetodrilus* have variable, irregular edges, the major lateral teeth of *Pseudolepetodrilus* gen. nov. have an even, outer slope without any notches or grooves.

###### Etymology.

The generic name means false (*pseudo*) *Lepetodrilus*, given its close physical resemblance to species of the genus *Lepetodrilus*.

##### ﻿ Pseudolepetodrilus costaricensis sp. nov.

Taxon classificationAnimaliaVetigastropodaLepetodrilidae

61CB524E-BC04-5225-BD28-36E00EC5EC20

https://zoobank.org/019D87DB-0CA1-4785-9E4E-7D9306AF0592

Fig. 9

###### Type material examined.

***Holotype***: Costa Rica • whole organism; ethanol-fixed; Original label: “*Pseudolepetodriluscostaricensis* holotype, 1, whole organism, AD4989, Costa Rica Margin, Jaco Scar, 9.11785, -84.8407, 1760 m, from tubeworms.”; SIO-BIC M22534. ***Paratypes***: Costa Rica • 1 specimen; same data as for holotype; Original label: “*Pseudolepetodriluscostaricensis* paratype, 1, whole organism, AD4989, Costa Rica Margin, Jaco Scar, 9.11785, -84.8407, 1760 m, from tubeworms.”; MZCR10673-01.

###### Type locality.

Costa Rica • Costa Rica Margin, Jaco Scar, 9.11785, -84.8407; hydrocarbon seep; tubeworms; 1,760 m; 4 November 2018; AT42-03 ALVIN Dive 4989 leg.

###### Other material examined.

Costa Rica • 4 specimen(s); Costa Rica Margin, Jaco Scar; 9.11785, -84.8407; 1,760 m; 4 November 2018; AT42-03 ALVIN Dive 4989 leg.; Tubeworm; EC10483.

###### Diagnosis.

*Pseudolepetodriluscostaricensis* sp. nov. can be diagnosed by their unique “wing-shaped” first major lateral tooth on their radula and through genetic characterization of the mitochondrial CO1 gene.

###### Description.

***Shell*** (Fig. 9G, H): Specimens exhibit patelliform shells with very small, truncated whorl at the posterior end of the shell. Holotype measures 3.7 mm in length, 2.8 mm in width, and 1.3 mm in maximum height. Shell roundness (width ÷ length) is ~ 0.75. Sinuous, concentric radial sculpturing present on shell with fine axial striations which intersect the radial sculpture to form very small, raised bumps. The aperture opening is ovate and unornamented. The aperture lip is thick and unornamented. The shell margin is flat. Posterior shell slope is flattened while the anterior shell slope is rounded. Shell apex is posteriorly shifted. Shell is robust with a thick, greenish brown periostracum covering the outer shell and wrapping over the aperture lip.

***Soft parts*** (Fig. 9I, M): Soft tissue is light greenish-to-yellowish in color. Mantle margin is thick and irregular and envelopes the body tissue. Three pairs of posterior epipodial tentacles are present. These tentacles descend in length, with the most anterior one being the longest and the most posterior one being the shortest. Posterior tentacles do not extend past the mantle margin. Two broad, fleshy, anterior tentacles are located approximately midway up and on either side of the foot margin. Two cephalic tentacles are present that are fleshy and triangular in shape and thicker than the epipodial tentacles. The mouth has a distinctive Y-shaped opening lacking thickened tissue. Elongated oral lappets are present. The penis originates from below the right cephalic tentacle. Operculum is absent.

***Radula*** (Fig. 9N, O): Rhipidoglossate radula. Rachidian teeth have very short shafts and sharp, triangular cusps. The anterior end of each cusp is flat while the pointed ends lack denticles. Rachidian teeth are flanked by one major lateral tooth on each side. Major laterals have broad, wing-shaped cusps that extend higher than the rachidian teeth. The outer edges of these cusps are serrated with ~ 16 short denticles. Three minor laterals follow which have long, sharp, triangular cusps whose outer edge is serrated with short denticles, but whose inner edges are not. The anterior edge of these minor laterals is slightly convex. The fourth, minor lateral teeth also have long, sharp, triangular cusps like the preceding three, but with serrations along both their inner and outer edges. Marginal teeth number ≥ 15 and exhibit rounded, spatulate cusps that are lined with ~ 40 denticles each. Denticles on each marginal tooth are elongated posteriorly and shorten as one moves anteriorly. Marginal cusps are located at the anterior end of a long, thin tooth shaft which connects to the radular ribbon at its base. Morphological transitions between major laterals, minor laterals, and marginal teeth are continuous.

###### Distribution.

*Pseudolepetodriluscostaricensis* sp. nov. is confirmed from the hydrocarbon seep sites “Jaco Scar” (9.12, -84.84) at the Pacific Costa Rica Margin. This species was sampled from tubeworms at 1,760 m depth.

###### Remarks.

Shells of this species notably do not narrow at their anterior ends, similar to *L.shannonae* (Warén and Bouchet 2009). Radulae most closely resembled those of *L.guaymasensis*. However, unlike this species, the central teeth of *P.costaricensis* are larger and lack denticles on their cusps. Further, their first lateral teeth have a shape that is distinct from *L.guaymasensis*, exhibiting an even, sloping outer ridge.

###### Etymology.

The species name *costaricensis* refers to the Pacific Costa Rica Margin, the geographic location where this species, and its genus, was first discovered.

#### ﻿Subclass Neomphaliones

**Family CocculinidaeDall 1882**

**Genus *Cocculina*Dall 1882**

##### ﻿ Cocculina methana sp. nov.

Taxon classificationAnimaliaVetigastropodaCocculinidae

730EE718-9358-5CC3-8B42-BA10C7AE124D

https://zoobank.org/C1481891-E0FF-4F55-8EE3-619ADE850CC4

Fig. 10

###### Type material examined.

***Holotype***: Costa Rica • whole organism; ethanol-fixed; Original label: “*Cocculinamethana* holotype, 1, whole organism, AD4924, Costa Rica Margin, Quepos Seep, 9.03174, -84.62158, 1408 m, from clams.”; SIO-BIC M22533. ***Paratypes***: Costa Rica • 1 specimen; same data as for holotype; Original label: “*Cocculinamethana* paratype, 1, whole organism, AD4924, Costa Rica Margin, Quepos Seep, 9.03174, -84.62158, 1408 m, from clams.”; MZCR10672-01.

###### Type locality.

Costa Rica • Costa Rica Margin, Quepos Seep, 9.03174, -84.62158; hydrocarbon seep; clams; 1,408 m; 7 June 2017; AT37-13 ALVIN Dive 4924 leg.

###### Other material examined.

Costa Rica • 4 specimens; Costa Rica Margin, Quepos Seep; 9.03174, -84.62158; 1,408 m; 7 June 2017; AT37-13 ALVIN Dive 4924 leg.; Clams; Erik Cordes Personal Collection (EC) 5752 • 2 specimen(s); Costa Rica Margin, Mound 12; 8.929983333, -84.31167667; 992 m; 20 October 2018; AT42-03 ALVIN Dive 4974 leg.; Bone, SIO-BIC M16788 • 3 specimen(s); Costa Rica Margin, Jaco Scar; 9.11562, -84.84005; 1,908 m; 28 May 2017; AT37-13 ALVIN Dive 4913 leg.; Wood, SIO-BIC M16149 • 15 specimen(s); Costa Rica Margin, Jaco Scar; 9.1193, -84.84277; 1,854 m; 31 May 2017; AT37-13 ALVIN Dive 4916 leg.; Tubeworm, SIO-BIC M16171 • 30 specimen(s); Costa Rica Margin, Quepos Seep; 9.0303, -84.623; 1,433 m; 1 March 2009; AT15-44 ALVIN Dive 4508 leg.; SIO-BIC M12024 • 3 specimen(s); Costa Rica Margin, Jaco Scar; 9.1172, -84.8417; 1,866 m; 3 March 2009; AT15-44 ALVIN Dive 4509 leg.; SIO-BIC M12037 • 6 specimen(s); Costa Rica Margin, Mound Jaguar; 9.651755802, -85.88211866; 2,000 m; 25 January 2019; FK19-0106 SUBASTIAN Dive 230 leg.; Wood, SIO-BIC M17106 • 3 specimen(s); Costa Rica Margin, Mound Jaguar; 9.65876081, -85.88259157; 1,896 m; 25 January 2019; FK19-0106 SUBASTIAN Dive 230 leg.; Wood, SIO-BIC M17105.

###### Diagnosis.

*Cocculinamethana* sp. nov. may be diagnosed by its distinct golden-brown periostracum. It may be most reliably distinguished from its sister species, *Cocculinajaponica*, through mitochondrial CO1 barcoding.

###### Description.

***Shell*** (Fig. 10A–C): Specimens exhibit uncoiled, patelliform shells. Holotype measures 3.4 mm in length, 2.3 mm in width, and 1.7 mm in maximum height. Shell roundness (width ÷ length) is ~ 0.66. Fine, concentric, radial sculpturing present on shell. The aperture opening is ovate and unornamented. The aperture lip is thin, fragile, and unornamented. The shell margin is flat. Posterior shell slope is flattened while the anterior shell slope is rounded. Shell apex is posteriorly shifted. Protoconch is unknown. Shell is robust with a thick, greenish brown periostracum covering the outer shell and wrapping over the aperture lip.

***Soft parts*** (Fig. 10C): Soft tissue is light yellow to white in color. Mantle margin is thin and irregular. One pair of posterior epipodial tentacles present. Posterior tentacles are thin, elongated, and do not taper in width towards their distal ends. Two, short, blunt cephalic tentacles are present that are slightly thicker than the epipodial tentacles. The mouth has well-developed oral lappets surrounding a starburst-shaped oral opening. External reproductive structures were not observed. Foot margin is ovate and slightly irregular. Operculum is absent.

***Radula*** (Fig. 10I–K): Radula is rhipidoglossate. Rachidian teeth are highly diminished, lacking cusps; The rachidian teeth form a continuous, raised ridge down the center of the radula. Rachidian are flanked by three major lateral teeth on each side. Lateral teeth have spatulate cusps that decrease in size from the first to third tooth. First major lateral teeth are the broadest, having 6–8 rounded denticles on their cusps. Second major lateral teeth are slightly thinner, having 3–5 denticles on their cusps. Finally, the third major laterals are thinner than the other two, and have two or fewer denticles on their cusps. These three major laterals are followed by one minor lateral tooth, which is broader than any of the other teeth preceding it. This minor lateral tooth has a short cusp that is angled outwards with four or five blunt, rounded denticles. Each minor lateral tooth has one or two short denticles on their innermost side (raised the highest), followed by one broad, elongated denticle, and finally followed by another short denticle on its outermost, lowest side. Two sets of numerous, marginal teeth follow, set at different angles. Sets of inner marginal teeth are more or less parallel to the radular ribbon, and number 10–12 teeth. Each tooth has a very thin and long tooth shaft (thinner than any of the preceding teeth) and a spatulate cusp with 5–7 short, rounded denticles. Sets of outer marginals are set at ~ 45° angle to the radular ribbon, and number between 15–20 teeth. These outer marginals also have a thin and long tooth shaft with spatulate cusps. These cusps, however, are decorated with ~ 24 thin, bristle-like denticles (~ 12 on each side of the cusp).

###### Distribution.

*Cocculinamethana* sp. nov. is confirmed from the hydrocarbon seep sites Quepos Seep (9.03, -84.62), Mound 12 (8.93, -84.31), Jaco Scar (9.12, -84.84), and Mound Jaguar (9.66, -85.88) at the Pacific Costa Rica Margin. This species was sampled from clam shells, wood, tubeworms, and bone between 1,408–2,000 m depth. These are among the deepest-known *Cocculina*.

###### Remarks.

The shells of *Cocculinamethana* sp. nov. most closely resemble those of *C.japonica* (Dall 1907). Radulae of these specimens most closely resembled that of *C.cowani* (McLean 1987) but with distinct central teeth that form a narrow, defined ridge down the center of the radula (Fig. 9J). The shell apex of this species notably lacks the hooked “sail fin” appearance of other *Cocculina* species. The periostracum of these specimens was observed to significantly corrode with prolonged ethanol preservation (Fig. 10G, H). This should be considered when examining museum specimens.

###### Etymology.

The species name *methana* refers to the occurrence of this species at a hydrocarbon seep site. This habitat type is notable, as all other known species of *Cocculina* occur at either inactive hydrothermal vents or organic falls.

## ﻿Discussion

The present study aimed to investigate the diversity of gastropod limpets at the Pacific Costa Rica Margin (CRM) hydrocarbon seeps. Given the CRM’s unique geographic situation among multiple oceanic currents and its separation from other chemosynthetic regions, it was hypothesized that this region would host species that were related to, but distinct from, nearby chemosynthesis-based ecosystems. Using the informative genetic loci CO1 and H3, as well as shell and radular characters, four species and one new genus across three gastropod subclasses were identified from more than 4,000 limpet specimens. This study also confirmed the presence of *Lepetodrilusguaymasensis* at the CRM and expands the known range of *Pyropeltacorymba* southward to include CRM hydrocarbon seeps.

### ﻿New species

*Bathyacmaealevinae* sp. nov. found at the CRM is notably the first of its genus to be confirmed in the Eastern Pacific; all other species appear to be endemic to the Western Pacific (e.g., Okutani et al. 1992; Sasaki 2003; Zhang and Zhang 2020; Chen and Sigwart 2023). Thus, the present study expands the known range of this genus across the Pacific Ocean. This CRM species is most notable in its exhibiting distinct ecotypes depending on the substrate from which it was sampled. While studies of other marine gastropod groups have identified substrate-dependent morphology as being the result of disruptive selection exerted by predators (Hollander and Butlin 2010), other chemosynthesis-based gastropods have been found to display similar patterns of phenotypic plasticity across these or similar substrates (e.g., *Lepetodrilus* (Chen and Watanabe 2020), *Bathyacmaea* (Chen et al. 2019)). In agreement with previous studies of *Bathyacmaea*, little to no genetic distinction between our distinct morphotypes was found (Chen et al. 2019; Zhang and Zhang 2020). These previous findings support our conclusion of a single, phenotypically plastic species at the CRM. Despite these well-documented phenotypic differences across substrate, however, little is yet known approximately the functional purpose of these changes, or what metabolic drivers are behind it.

Another group exhibiting highly variable and confounding shells is the genus *Paralepetopsis*. Individuals within this genus were highly cryptic, with little to no discernible features with which to distinguish the species under examination from one another. There also appears to be no clear environmental separation within this group, with three of the five genetic clades identified being found at both the shallower site Mound 12 and the deeper site Jaco Scar (Table 1). All but one clade was identified from both mussels and tubeworms (Table 1). Furthermore, *Paralepetopsis*, as a genus, was present at all four sites examined in this study. Thus, the genetic diversity documented in this investigation was difficult to predict. Such sympatric speciation may be the result of cryptic mate choice or niche divergence that has not been fully characterized by the variables currently under investigation. Regardless, our investigation supports genetic barcoding as the most reliable way to identify *Paralepetopsis* species from the CRM. Furthermore, as more than 1,300 individuals from this genus were collected, it is highly likely that even more cryptic species may be hidden within this genus that remain to be described.

The new species *Paralepetopsisvariabilis* sp. nov. from the CRM seems to have been first collected from the Pescadero Basin in the Gulf of California, with one representative sequence on GenBank (KY581541, Fig. 12A). This species is described herein with permission from the corresponding author of that sequence (pers. corr. Shannon Johnson). This connectivity is notable, as the CRM and the Pescadero Basin are located more than 3,500 km apart from one another, yet may be connected through the California or West Mexican currents (Kessler 2006). Our species most closely resembled *P.tunnicliffae*; unfortunately, no published barcodes for CO1 nor H3 exist for *P.tunnicliffae* with which to compare our specimens. However, *P.tunnicliffae* was first described from hydrothermal vents at the Juan de Fuca Ridge, which may be consequential; species turnover and genetic structuring has been observed between hydrothermal vent fields in the Northeastern Pacific and the equatorial Eastern Pacific across multiple invertebrate taxa (Tunnicliffe et al. 1996) including *Lepetodrilus* limpets (Matabos and Jollivet 2019), aplacophorans (Scheltema 2008), and alvinellid worms (Chevaldonne et al. 2002). Such differences are ascribed to the subduction of the Farallon Plate beneath the North American Plate approximately 28 million years ago (Tunnicliffe et al. 1996; Vrijenhoek 2013). Furthermore, the distinction between vent and seep environments also tends to correlate with species turnover (e.g., Tunnicliffe et al. 1996). While we propose that this geological context, along with morphological differences, makes it unlikely that our Costa Rican specimens are *P.tunnicliffae*, genetic characterization of, and comparison with, *P.tunnicliffae* would still be warranted.

This study identified a single species of *Cocculina* limpets from the CRM, *Cocculinamethana* sp. nov. (Table 1). While species in this genus are typically found at organic falls, such as wood or whale carcasses in both shallow and deep water, the species identified here was found at the hydrocarbon seep site Quepos Seep, which is a soft-bottom, low productivity mud volcano at the CRM. The relatively low level of hydrocarbons and sulfides at this site may have made it ideal for the settlement of *Cocculina* limpets. Furthermore, the holotype of this species was obtained from a “Bushmaster” quantitative sample that also contained foundational seep species such as the mussel *Bathymodiolusbillschneideri* McCowin, Feehery & Rouse, 2020 and the tubeworm *Lamellibrachia* sp. (Webb 1969), suggesting the presence, tolerance, and potential reliance on hydrocarbon seepage in its environment.

*Pyropeltacorymba* and *Lepetodrilusguaymasensis* were the two known species identified from the CRM. *Lepetodrilusguaymasensis* has been previously collected and genetically characterized from both the Guaymas Basin (from which it was originally described) and the CRM. Thus, it was not surprising that this species was found here and displayed the expected genetic affinity to these previously obtained sequences. *Pyropeltacorymba*, however, has not been identified from the CRM before, and thus these represent novel records of occurrence for this group. Specifically, *P.corymba* from the CRM represent the most southerly population of this species known to date. While *P.corymba* has been morphologically identified from vents and seeps in the Gulf of California (McLean 1992) as well as from whale bone in the Catalina Basin (McLean and Haszprunar 1987), there are no associated gene sequences that have been published from these populations. Thus, it is impossible to assess the relatedness between our samples and these more northerly populations.

Finally, this study identifies a new genus within the family Lepetodrilidae, *Pseudolepetodrilus* gen. nov. The other genera within this family (*Lepetodrilus*, *Pseudorimula*, *Gorgoleptis*, and *Clypeosectus*) are all endemic to chemosynthesis-based ecosystems and have been extensively studied over the past 50 years. This is particularly true for *Lepetodrilus*, which are highly abundant and well-characterized from the East Pacific Rise hydrothermal vents, in particular. Therefore, the identification of a wholly new genus within this family was unexpected. These specimens were relatively rare in our collections, having only been collected during one dive at Jaco Scar with a total of ten individuals identified. Morphologically, this new genus undoubtedly most closely resembles *Lepetodrilus* out of the Lepetodrilids. However, genetically, it is placed as sister to *Pseudorimula* (Fig. 14A), and has three pairs of posterior epipodial tentacles, like *Cylpeosectus*. Further research is needed to understand when and where this new genus likely originated, and whether it is represented by any additional species elsewhere.

### ﻿Data availability

While results from our genetic investigations support the species herein described from the CRM, we nonetheless draw attention to the relatively small sample sizes of several genera investigated. *Pyropelta*, for instance, is only represented on GenBank by three published and one unpublished mitochondrial CO1 sequence outside of the novel sequences herein generated from the CRM. Similarly, *Paralepetopsis* is represented by just seven published mitochondrial gene sequences outside of the novel sequences generated from the CRM. Several species that were close morphological matches to our own, such as *Bathyacmaeakanesunosensis*, *Paralepetopsistunnicliffae*, *Pyropeltacorymba*, and *Pyropeltamusaica*, had no associated gene sequences on public repositories, precluding genetic comparison. This shortage of sequences may lead to an overestimation of exclusivity and may mask consequential connections between the CRM and other regions. These data deficiencies highlight one of the core challenges of conducting deep-sea taxonomic work: Genetic samples are often scarce. This scarcity may arise from a lack of specimens (e.g., the general inaccessibility of these environments, the differing sampling regimes employed by expeditions), a lack of useable genetic material (e.g., preserved specimens fixed in formalin), or a lack of taxonomic work being conducted (e.g., personal, private, and museum collections awaiting genetic characterization). Furthermore, the present article only describes deceased specimens, as notes and photographs of live specimens were not obtained prior to ethanol preservation. Information regarding the morphological characters and behaviors of live specimens thus represents an avenue for future characterization and research.

## ﻿Conclusions

This study conducted genetic and morphological investigations of limpets from the hydrocarbon seeps at the CRM. These investigations found support for the novelty of several limpet species at the CRM including *Bathyacmaealevinae* sp. nov., *Paralepetopsisvariabilis* sp. nov., *Pseudolepetodriluscostaricensis* gen. et sp. nov., and *Cocculinamethana* sp. nov. This study also presents new occurrence records for the known species *Lepetodrilusguaymasensis* and *Pyropeltacorymba*. This study contributes to the growing body of knowledge surrounding the biodiversity of the deepwater off Costa Rica. Future investigations examining the diversity of other deep-sea animal groups at the Costa Rica Margin may reveal additional novel species that should be of interest to regional and global conservation efforts.

## Supplementary Material

XML Treatment for Bathyacmaea levinae

XML Treatment for Paralepetopsis variabilis

XML Treatment for Pyropelta corymba

XML Treatment for Lepetodrilus guaymasensis

XML Treatment for Pseudolepetodrilus

XML Treatment for Pseudolepetodrilus costaricensis

XML Treatment for Cocculina methana

## ﻿Additional information

### Author ORCIDs

Melissa J. Betters https://orcid.org/0000-0002-8975-257X

Jorge Cortés https://orcid.org/0000-0001-7004-8649

Erik E. Cordes https://orcid.org/0000-0002-6989-2348

### Data availability

Gene sequences generated in this study are accessible on GenBank under the accession numbers OQ644569–OQ644631 and Q658576–OQ658595. Occurrence records may be accessed from the Global Biodiversity Information Facility at https://ipt.pensoft.net/resource?r=crm_limpets&v=1.0. This publication and associated nomenclatural acts are registered with Zoobank at LSID urn:lsid:zoobank.org:pub:487E305B-E2EF-4D96-8940-4C4141C0BA91.

## Supplementary materials

Supplementary material 1 PCR Reaction conditions used to successfully amplify loci Data type docx Explanation note PCR components were used at the following concentrations: MgCl (25 mM), BSA (10 mg/mL), DNTPs (2.5 mM each), Primers (10 uM each). PCR Protocols are as follows: 1 = { 4 min at 94 °C, 35 × (1 min at 95 °C, 1 min at 40 °C, 1.5 min at 72 °C), 7 min at 72 °C}, 2 = { 5 min at 94 °C, 35 × (30 sec at 94 °C, 1 min at 45 °C, 1 min at 72 °C), 5 min at 72 °C}, 3 = same as 2 but 30 cycles instead of 35, 4 = {2 min at 94 °C, 40 × (20 sec at 94 °C, 20 sec at 55 °C, 1 min at 68 °C)}, 5 = {2 min at 94 °C, 40 × (20 sec at 94 °C, 20 sec at 65 °C, 1 min at 72 °C)}. Accession numbers refer to identities within NCBI GenBank. “” = Same value as the cell above. https://binary.pensoft.net/file/1150586This dataset is made available under the Open Database License (http://opendatacommons.org/licenses/odbl/1.0/). The Open Database License (ODbL) is a license agreement intended to allow users to freely share, modify, and use this Dataset while maintaining this same freedom for others, provided that the original source and author(s) are credited.Melissa J. Betters, Jorge Cortés, Erik E. Cordes
