# Supplementary material for: ﻿New species and records of limpets (Mollusca, Gastropoda) from the Pacific Costa Rica Margin
Source: Zookeys. 2024 Oct 9;1214:281–324. doi: 10.3897/zookeys.1214.128594 (PMC11491734; doi:10.3897/zookeys.1214.128594)
Supplement: Supplementary material 1 — PCR Reaction conditions used to successfully amplify loci [file zookeys-1214-281_article-128594__-s001.docx]

| **Accession** | **Locus** | **Primers** | **Buffer** | **Taq** | **Buffer** | **Taq** | **MgCl** | **BSA** | **DNTP** | **Primer (F)** | **Primer (R)** | **H2O** | **DNA** | **Protocol** |
| --- | --- | --- | --- | --- | --- | --- | --- | --- | --- | --- | --- | --- | --- | --- |
| OQ644569 | CO1 | LCO1490 / HCO2198 | 10X Econotaq without MgCl | 10 U of EconoTaq | 2.5 | 0.25 | 2 | 1 | 2.5 | 1 | 1 | 12.75 | 2 | 1 |
| OQ644571 | CO1 | “” | “” | “” | “” | “” | “” | “” | “” | “” | “” | “” | “” | “” |
| OQ644572 | CO1 | “” | “” | “” | “” | “” | “” | “” | “” | “” | “” | “” | “” | “” |
| OQ644573 | CO1 | “” | “” | “” | “” | “” | “” | “” | “” | “” | “” | “” | “” | “” |
| OQ644574 | CO1 | “” | “” | “” | “” | “” | “” | “” | “” | “” | “” | “” | “” | “” |
| OQ644576 | CO1 | “” | “” | “” | “” | “” | “” | “” | “” | “” | “” | “” | “” | “” |
| OQ644579 | CO1 | “” | “” | “” | “” | “” | “” | “” | “” | “” | “” | “” | “” | “” |
| OQ644580 | CO1 | “” | “” | “” | “” | “” | “” | “” | “” | “” | “” | “” | “” | “” |
| OQ644581 | CO1 | “” | “” | “” | “” | “” | “” | “” | “” | “” | “” | “” | “” | “” |
| OQ644582 | CO1 | “” | “” | “” | “” | “” | “” | “” | “” | “” | “” | “” | “” | “” |
| OQ644583 | CO1 | “” | “” | “” | “” | “” | “” | “” | “” | “” | “” | “” | “” | “” |
| OQ644584 | CO1 | “” | “” | “” | “” | “” | “” | “” | “” | “” | “” | “” | “” | “” |
| OQ644585 | CO1 | “” | “” | “” | “” | “” | “” | “” | “” | “” | “” | “” | “” | “” |
| OQ644586 | CO1 | “” | “” | “” | “” | “” | “” | “” | “” | “” | “” | “” | “” | “” |
| OQ644589 | CO1 | “” | “” | “” | “” | “” | “” | “” | “” | “” | “” | “” | “” | “” |
| OQ644590 | CO1 | “” | “” | “” | “” | “” | “” | “” | “” | “” | “” | “” | “” | “” |
| OQ644591 | CO1 | “” | “” | “” | “” | “” | “” | “” | “” | “” | “” | “” | “” | “” |
| OQ644592 | CO1 | “” | “” | “” | “” | “” | “” | “” | “” | “” | “” | “” | “” | “” |
| OQ644593 | CO1 | “” | “” | “” | “” | “” | “” | “” | “” | “” | “” | “” | “” | “” |
| OQ644594 | CO1 | “” | “” | “” | “” | “” | “” | “” | “” | “” | “” | “” | “” | “” |
| OQ644595 | CO1 | “” | “” | “” | “” | “” | “” | “” | “” | “” | “” | “” | “” | “” |
| OQ644596 | CO1 | “” | “” | “” | “” | “” | “” | “” | “” | “” | “” | “” | “” | “” |
| OQ644598 | CO1 | “” | “” | “” | “” | “” | “” | “” | “” | “” | “” | “” | “” | “” |
| OQ644599 | CO1 | “” | “” | “” | “” | “” | “” | “” | “” | “” | “” | “” | “” | “” |
| OQ644600 | CO1 | “” | “” | “” | “” | “” | “” | “” | “” | “” | “” | “” | “” | “” |
| OQ644602 | CO1 | “” | “” | “” | “” | “” | “” | “” | “” | “” | “” | “” | “” | “” |
| OQ644603 | CO1 | “” | “” | “” | “” | “” | “” | “” | “” | “” | “” | “” | “” | “” |
| OQ644604 | CO1 | “” | “” | “” | “” | “” | “” | “” | “” | “” | “” | “” | “” | “” |
| OQ644605 | CO1 | “” | “” | “” | “” | “” | “” | “” | “” | “” | “” | “” | “” | “” |
| OQ644606 | CO1 | “” | “” | “” | “” | “” | “” | “” | “” | “” | “” | “” | “” | “” |
| OQ644607 | CO1 | “” | “” | “” | “” | “” | “” | “” | “” | “” | “” | “” | “” | “” |
| OQ644608 | CO1 | “” | “” | “” | “” | “” | “” | “” | “” | “” | “” | “” | “” | “” |
| OQ644609 | CO1 | “” | “” | “” | “” | “” | “” | “” | “” | “” | “” | “” | “” | “” |
| OQ644610 | CO1 | “” | “” | “” | “” | “” | “” | “” | “” | “” | “” | “” | “” | “” |
| OQ644611 | CO1 | “” | “” | “” | “” | “” | “” | “” | “” | “” | “” | “” | “” | “” |
| OQ644612 | CO1 | “” | “” | “” | “” | “” | “” | “” | “” | “” | “” | “” | “” | “” |
| OQ644616 | CO1 | “” | “” | “” | “” | “” | “” | “” | “” | “” | “” | “” | “” | “” |
| OQ644617 | CO1 | “” | “” | “” | “” | “” | “” | “” | “” | “” | “” | “” | “” | “” |
| OQ644618 | CO1 | “” | “” | “” | “” | “” | “” | “” | “” | “” | “” | “” | “” | “” |
| OQ644627 | CO1 | “” | “” | “” | “” | “” | “” | “” | “” | “” | “” | “” | “” | “” |
| OQ644628 | CO1 | “” | “” | “” | “” | “” | “” | “” | “” | “” | “” | “” | “” | “” |
| OQ644629 | CO1 | “” | “” | “” | “” | “” | “” | “” | “” | “” | “” | “” | “” | “” |
| OQ644630 | CO1 | “” | “” | “” | “” | “” | “” | “” | “” | “” | “” | “” | “” | “” |
| OQ644597 | CO1 | LCO1490 / HCO2198 | 10X Econotaq *WITH* MgCl | 10 U of EconoTaq | 2.5 | 0.25 | 0 | 1 | 2.5 | 1 | 1 | 14.75 | 2 | 1 |
| OQ644601 | CO1 | “” | “” | “” | “” | “” | “” | “” | “” | “” | “” | “” | “” | “” |
| OQ644619 | CO1 | “” | “” | “” | “” | “” | “” | “” | “” | “” | “” | “” | “” | “” |
| OQ644620 | CO1 | “” | “” | “” | “” | “” | “” | “” | “” | “” | “” | “” | “” | “” |
| OQ644622 | CO1 | “” | “” | “” | “” | “” | “” | “” | “” | “” | “” | “” | “” | “” |
| OQ644623 | CO1 | “” | “” | “” | “” | “” | “” | “” | “” | “” | “” | “” | “” | “” |
| OQ644624 | CO1 | “” | “” | “” | “” | “” | “” | “” | “” | “” | “” | “” | “” | “” |
| OQ644625 | CO1 | “” | “” | “” | “” | “” | “” | “” | “” | “” | “” | “” | “” | “” |
| OQ644626 | CO1 | “” | “” | “” | “” | “” | “” | “” | “” | “” | “” | “” | “” | “” |
| OQ644570 | CO1 | LCO1490 / HCO2198 | 10X Econotaq without MgCl | 10 U of EconoTaq | 2.5 | 0.25 | 2 | 1 | 2.5 | 1 | 1 | 12.75 | 2 | 2 |
| OQ644587 | CO1 | LCO1490 / HCO2198 | 10X Econotaq without MgCl | 10 U of EconoTaq | 2.5 | 0.25 | 2 | 1 | 2.5 | 1 | 1 | 12.75 | 2 | 3 |
| OQ644588 | CO1 | “” | “” | “” | “” | “” | “” | “” | “” | “” | “” | “” | “” | “” |
| OQ644575 | CO1 | LCO1490 / HCO2198 | Accustart II PCR ToughMix | NA | 12.5 | 0 | 0 | 0 | 0 | 1.25 | 1.25 | 5 | 5 | 4 |
| OQ644577 | CO1 | “” | “” | “” | “” | “” | “” | “” | “” | “” | “” | “” | “” | “” |
| OQ644613 | CO1 | “” | “” | “” | “” | “” | “” | “” | “” | “” | “” | “” | “” | “” |
| OQ644614 | CO1 | “” | “” | “” | “” | “” | “” | “” | “” | “” | “” | “” | “” | “” |
| OQ644615 | CO1 | “” | “” | “” | “” | “” | “” | “” | “” | “” | “” | “” | “” | “” |
| OQ644621 | CO1 | “” | “” | “” | “” | “” | “” | “” | “” | “” | “” | “” | “” | “” |
| OQ658576 | H3 | HN3F/R | Accustart II PCR ToughMix | NA | 12.5 | 0 | 0 | 0 | 0 | 1.25 | 1.25 | 5 | 5 | 4 |
| OQ658578 | H3 | “” | “” | “” | “” | “” | “” | “” | “” | “” | “” | “” | “” | “” |
| OQ658579 | H3 | “” | “” | “” | “” | “” | “” | “” | “” | “” | “” | “” | “” | “” |
| OQ658589 | H3 | “” | “” | “” | “” | “” | “” | “” | “” | “” | “” | “” | “” | “” |
| OQ658590 | H3 | “” | “” | “” | “” | “” | “” | “” | “” | “” | “” | “” | “” | “” |
| OQ658582 | H3 | H3F/R | ^ | NA | 12.5 | 0 | 0 | 0 | 0 | 1.25 | 1.25 | 5 | 5 | 4 |
| OQ658583 | H3 | “” | “” | “” | “” | “” | “” | “” | “” | “” | “” | “” | “” | “” |
| OQ658584 | H3 | “” | “” | “” | “” | “” | “” | “” | “” | “” | “” | “” | “” | “” |
| OQ658585 | H3 | “” | “” | “” | “” | “” | “” | “” | “” | “” | “” | “” | “” | “” |
| OQ658586 | H3 | “” | “” | “” | “” | “” | “” | “” | “” | “” | “” | “” | “” | “” |
| OQ658587 | H3 | “” | “” | “” | “” | “” | “” | “” | “” | “” | “” | “” | “” | “” |
| OQ658588 | H3 | “” | “” | “” | “” | “” | “” | “” | “” | “” | “” | “” | “” | “” |
| OQ658592 | H3 | “” | “” | “” | “” | “” | “” | “” | “” | “” | “” | “” | “” | “” |
| OQ658594 | H3 | “” | “” | “” | “” | “” | “” | “” | “” | “” | “” | “” | “” | “” |
| OQ658595 | H3 | “” | “” | “” | “” | “” | “” | “” | “” | “” | “” | “” | “” | “” |
| OQ658581 | H3 | H3F/R | ^ | NA | 12.5 | 0 | 0 | 0 | 0 | 1.25 | 1.25 | 5 | 5 | 5 |

**Supplementary Table 1.** PCR Reaction conditions used to successfully amplify loci. PCR components were used at the following concentrations: MgCl (25 mM), BSA (10 mg/mL), DNTPs (2.5 mM each), Primers (10 uM each). PCR Protocols are as follows: 1 = { 4 min at 94°C, 35 x (1 min at 95°C, 1 min at 40°C, 1.5 min at 72°C), 7 min at 72°C}, 2 = { 5 min at 94°C, 35 x (30 sec at 94°C, 1 min at 45°C, 1 min at 72°C), 5 min at 72°C}, 3 = same as 2 but 30 cycles instead of 35, 4 = {2 min at 94°C, 40 x (20 sec at 94°C, 20 sec at 55°C, 1 min at 68°C)}, 5 = {2 min at 94°C, 40 x (20 sec at 94°C, 20 sec at 65°C, 1 min at 72°C)}. Accession numbers refer to identities within NCBI GenBank. “” = Same value as the cell above.
